# ABHD4-2: a cardiolipin-selective phospholipase of the outer mitochondrial membrane

**DOI:** 10.1016/j.jlr.2026.101119

**Published:** 2026-08-06

**Authors:** Raja Narayanasamy, Matthew A. Sanders, Huamei Zhang, Marta Moreno-Torres, Vanesa D. Ramseyer, Hong-Guang Wei, Nils J. Færgeman, Christopher V. Kelly, James G. Granneman

**Affiliations:** 1 Center for Molecular Medicine and Genetics, Wayne State University School of Medicine, Detroit, MI, USA; 2 Department of Biochemistry and Molecular Biology, University of Southern Denmark, Odense M, Denmark; 3 Department of Physics and Astronomy, Wayne State University, Detroit, MI, USA; 4 Barber Center for Multiscale Systems Biology, Wayne State University, Detroit, MI, USA; 5 Center for Integrative Metabolic and Endocrine Research, Wayne State University School of Medicine, Detroit, MI, USA

**Keywords:** cardiolipin, ABHD4-2, outer mitochondrial membrane, cardiolipin hydrolase, phospholipase activity

## Abstract

Cardiolipin (CL) is a unique dimeric phospholipid essential for mitochondrial integrity and stress signaling. While most CL is present in the inner mitochondrial membranes (IMM), CL can be exposed to the outer mitochondrial membrane (OMM) under specific physiological and pathological conditions; however, the mechanisms regulating its metabolism at the OMM are poorly defined. Based on its striking structural similarity to cardiolipin deacylase 1 protein (Cld1p), a yeast CL hydrolase, we hypothesized that α/β-hydrolase domain-containing protein 4 (ABHD4) functions as a mammalian CL hydrolase. We identified two isoforms of mouse ABHD4 arising from alternative splicing: ABHD4-1 localizes to lipid droplets, whereas ABHD4-2 selectively targets mitochondria and is enriched in oxidative tissues. We demonstrate that ABHD4 selectively hydrolyzes CL in vitro, producing monolysocardiolipin and dilysocardiolipin. Site-directed mutagenesis identified catalytic serine residues as essential for enzymatic activity in ABHD4 and its *Drosophila* homolog, Pummelig. In contrast to Cld1p, which resides in the IMM, topological analyses indicate that ABHD4-2 and Pummelig-2 localize to the cytosolic face of the OMM, a conserved orientation that suggests a function distinct from classic acyl chain remodeling of CL. In brown adipocytes, ABHD4-2 overexpression reduces mitochondrial membrane potential in an activity-dependent manner. In silico analyses further reveal conservation of the catalytic triad across yeast, insect, and mammalian orthologs, supporting an evolutionarily conserved role for this enzyme family in CL metabolism. Together, these findings identify ABHD4-2 as an OMM-localized phospholipase with preferential CL hydrolase activity and define an isoform-specific pathway linking CL metabolism to mitochondrial stress responses.

Cardiolipin (CL) is a mitochondrial-enriched phospholipid that plays a central role in membrane organization and bioenergetic function (1, 2). Structurally, CL contains two phosphatidyl moieties linked by a central glycerol backbone, generating a lipid with four acyl chains. This unique architecture supports cristae membrane formation, stabilizes respiratory chain supercomplexes, and promotes efficient oxidative phosphorylation and ATP production (1, 3, 4). In general, CL is synthesized in the inner leaflet, where it is highly enriched and contributes to membrane organization (5). Alterations in the CL composition, distribution, or remodeling are associated with multiple pathologies, including Barth syndrome, cardiomyopathy, heart failure, neurodegenerative disorders, and metabolic disease (3, 6, 7). Although CL is predominantly localized to the inner membrane, a smaller but functionally significant pool is present at the outer mitochondrial membrane (OMM). At this location, CL represents a minor fraction of mitochondrial phospholipids and is thought to function as a signaling lipid in mitophagy, apoptosis, and mitochondrial quality control and can be redistributed to other cellular membranes during receptor-mediated apoptosis (8, 9, 10, 11).

CL acyl chain composition is remodeled on the inner mitochondrial membrane (IMM) through cycles of deacylation and reacylation (2, 5, 12). In yeast, this process is initiated by the IMM deacylase Cld1p (cardiolipin deacylase 1 protein) (13), which generates monolysocardiolipin (MLCL), followed by reacylation by tafazzin (6, 12). Loss of this enzyme impairs CL remodeling and is associated with impaired respiration, reduction in membrane potential, and increased sensitivity to oxidative stress (14, 15, 16). In higher eukaryotes, however, only a limited number of enzymes with analogous functions have been identified. Recent studies have identified α/β-hydrolase domain-containing protein 18 (ABHD18) as a Cld1p-related enzyme that catalyzes CL hydrolysis by the stepwise removal of its fatty acids to generate MLCL and dilysocardiolipin (DLCL) (17, 18). Other lipases, such as patatin-like phospholipase domain-containing protein 8/Ca2+-independent phospholipase A2γ, have been implicated in CL remodeling (19, 20), although CL metabolism at the OMM remains largely undefined.

CL exposed at the OMM has emerged as a key signaling platform that coordinates autophagy initiation, mitophagy, apoptotic complex assembly, mitochondrial dynamics, protein import, and broader cellular stress responses (8, 21, 22, 23, 24, 25). Despite this, the enzymes responsible for regulating signaling pools of CL at the OMM remain incompletely defined. Phylogenetic and structural analyses identified mammalian α/β-hydrolase domain-containing protein 4 (ABHD4) and its *Drosophila* homolog Pummelig as the closest paralogs to the yeast CL hydrolase Cld1p (13). Previous studies identified ABHD4 as an N-acyl phosphatidylethanolamine (NAPE) hydrolase linked to endocannabinoid signaling (26, 27). Subsequent work implicated ABHD4 in anoikis, a specialized form of programmed cell death triggered by loss of cell matrix attachment (28), as well as in adipocyte differentiation (29), independent of NAPE hydrolysis (30). Notably, ABHD4 retains conserved α/β-hydrolase architecture shared with Cld1p, supporting a potential role in CL metabolism. However, whether ABHD4 functions as a CL hydrolase, and how its splice variants contribute to this activity, remain unclear.

Here, we identify ABHD4 as a conserved mammalian CL hydrolase and define its relationship to yeast Cld1p and Pummelig. Alternative splicing generates two ABHD4 isoforms with distinct subcellular localizations. Both ABHD4 and Pummelig encode isoforms that exhibit CL hydrolase activity but differ in targeting. ABHD4-2 localizes to mitochondria, is enriched in oxidative tissues, and is exposed to the cytosolic face of the OMM as demonstrated by protease protection analysis. This localization positions ABHD4-2 to potentially access OMM associated CL pools. Biochemical and lipidomic analyses further show that ABHD4 catalyzes the sequential deacylation of CL to generate MLCL and DLCL. Although ABHD4 can hydrolyze multiple phospholipids, it exhibits preferential activity toward CL, and hydrolysis of other phospholipids is highly dependent on the presence of CL in the mixed membrane system. These findings identify ABHD4 as an OMM-localized phospholipase with preferential CL hydrolase activity and provide new insight into CL metabolism at the OMM.

## Materials and methods

### Reagents

All lipids, fluorescent lipid substrates, and mass spectrometry internal standards were obtained from Avanti Polar Lipids unless indicated otherwise. Proteinase K (recombinant; Cat. No. EO0491) was obtained from Thermo Fisher Scientific. Rabbit polyclonal ABHD4 antibodies were obtained from Thermo Fisher Scientific (Cat. No. PA5-75867) and Sigma-Aldrich (ABHD4, center antigen; Cat. No. SAB1307058), rabbit monoclonal TOM20 antibody (D8T4N) from Cell Signaling Technology (Cat. No. 42406), mouse monoclonal NDUFS1 antibody from Santa Cruz Biotechnology (Cat. No. sc-271510), mouse monoclonal cytochrome c antibody from BD Pharmingen (Cat. No. 556433), mouse monoclonal His-tag antibody from Thermo Fisher Scientific (Cat. No. MA1-21315), and HRP-conjugated secondary antibodies from Jackson ImmunoResearch. HaloTag ligand (Cat. No. G3221) was obtained from Promega. LipidTOX™ Deep Red neutral lipid stain (Cat. No. H34477) and MitoTracker™ Deep Red FM (Cat. No. M22426) were obtained from Thermo Fisher Scientific. Silica gel 60 F254 thin-layer chromatography (TLC) aluminum plates (Cat. No. 1.05554.0001) were obtained from Merck. Analytical-grade solvents used for lipid extraction were obtained from Sigma-Aldrich. All other analytical-grade reagents were purchased from Thermo Fisher Scientific or Sigma-Aldrich.

### Mice and tissue lysates

Adult male C57BL/6J mice (12 weeks old) were maintained at 22°C under a 12 h light/dark cycle with ad libitum access to chow and water. Mice were euthanized by CO_2_ asphyxiation, and tissues were rapidly excised, snap-frozen in liquid N_2_, and stored at −80°C until use. Tissue samples were homogenized (20 mg/ml) in RIPA buffer (50 mm Tris-HCl, pH 7.4, 150 mm NaCl, 1% NP-40, 0.1% SDS) supplemented with protease inhibitors. Protein concentrations were determined using a BCA assay.

### Study approval

Animal procedures used in this study were conducted in accordance with protocols approved by the Institutional Animal Care and Use Committee at Wayne State University.

### Immunoblotting

Mouse tissues were homogenized in lysis buffer, and protein concentrations were determined by standard assays. Equal amounts of protein were resolved by SDS-PAGE and transferred to membranes for immunoblotting. Signals were developed using SuperSignal West Dura Substrate (Thermo Fisher Scientific; Cat. No. 34076) and captured using an Azure Biosystems c600 imaging system. Lysates from COS-7 cells transiently expressing ABHD4 isoforms were included as controls. Immunoblots were detected using the indicated primary antibodies followed by HRP-conjugated secondary antibodies. Subcellular fractions of brown adipose tissue (BAT) were prepared by differential centrifugation (31). Protein concentrations were determined using standard assays.

### Cell culture

COS-7 cells were cultured in DMEM supplemented with 10% fetal bovine serum and penicillin-streptomycin (100 U/ml, 100 μg/ml) at 37°C, 5% CO_2_. Cells were then transiently transfected with the indicated plasmids using Lipofectamine and Plus Reagent (Invitrogen) following the manufacturer’s instructions. Brown adipocyte (BA) cells were cultured in DMEM supplemented with 10% fetal bovine serum and penicillin-streptomycin at 37°C in 5% CO_2_.

### Confocal microscopy

Prior to imaging, transfected cells on coverslips were lipid loaded for 16–20 h with 200 μm oleic acid, then stained for 30 min at 37°C with LipidTOX Deep Red (1:1,000 dilution) and 100 nm MitoTracker Deep Red. Cells were then fixed with 4% paraformaldehyde in PBS. Imaging was performed using an Andor Dragonfly spinning disk confocal microscope equipped with a 63× oil-immersion objective (NA 1.4). Images were acquired using Fusion software (Oxford Instruments Andor, Belfast, Northern Ireland) under identical exposure settings for all experimental conditions. Z-stacks were collected as needed, and maximum intensity projections were generated using ImageJ.

### Proteinase K protection assay

Mitochondria were isolated from homogenized BAT or transfected COS-7 cells by differential centrifugation in homogenization buffer (225 mm mannitol, 75 mm sucrose, 10 mm HEPES-KOH, 1 mm EDTA, pH 7.4). Mitochondria were then treated with proteinase K (0–1 mg/ml) for 15 min at 37°C. Reactions were stopped by incubating samples on ice and the addition of 0.1 volume of 200 mm PMSF, prior to analysis by SDS-PAGE. A membrane-permeabilized control was treated with 0.5 mg/ml proteinase K in the presence of 0.5% Triton X-100. Immunoblots were probed with antibodies against ABHD4, TOM20 (OMM marker), cytochrome c [intermembrane space (IMS) marker], and NADH:ubiquinone oxidoreductase core subunit S1 (NDUFS1) (IMM marker), as indicated.

### Recombinant expression and purification

Bacmids encoding His-tagged ABHD4 isoforms or Pummelig isoforms were obtained by transformation of *Escherichia coli DH10Bac* cells (Invitrogen) with pFastBac vectors containing the respective His-tagged protein. Catalytic mutant constructs were generated using standard molecular cloning methods and verified by Sanger sequencing. Baculovirus was obtained by bacmid transfection of *Spodoptera frugiperda* (Sf9) cells using CellFectin reagent (Invitrogen), followed by virus amplification in Sf9 cells. For protein purification, High Five cells were infected with amplified baculovirus and then collected 24–48 h post-infection. High Five cells were then lysed in IB buffer (10 mm Hepes, pH 7.2, 140 mm KCl, 6 mm NaCl, 1 mm MgCl_2_) containing 20 μg/ml leupeptin and pepstatin A by sonication. After centrifugation (15,000g, 10 min, 4°C), proteins were purified from lysate supernatants using Takara Talon Co^2+^ affinity resin.

### In vitro cardiolipin lipase assay and free fatty acid quantification

The procedure for measuring ABHD4 cardiolipin lipase activity was adapted from Ipsaro *et al*. (32) with modifications. Lipid vesicle mixtures were prepared with and without bovine heart CL, dried under N_2_, and resuspended in 375 μl of 10 mm Hepes/100 mm NaCl by sonication. After sonication, 125 μl of 20% BSA in 10 mm Hepes/100 mm NaCl was added to the vesicles. Reaction mixtures consisted of 80 μl of buffer containing 50 mm Hepes (pH 7.4), 80 mm KCl, 3 mm MgCl_2_, 2 mm CaCl_2_, and 1 mm DTT, along with 10 μl of lipid vesicles and 10 μl (2 μg) of partially purified ABHD4 protein. For reactions with CL vesicles, final lipid concentrations were 80 μm 1,2-dioleoyl-sn-glycero-3-phosphocholine (DOPC), 80 μm heart CL, 40 μm phosphatidylserine (PS) (18:0/18:1), and 40 μm phosphatidylethanolamine (PE) (18:0/18:1). For reactions without CL, final lipid concentrations were 120 μm DOPC, 60 μm PS, and 60 μm PE. After ∼2.5 h at 37°C, free fatty acids (FFAs) were measured using a NEFA kit (Wako Diagnostics) adapted to Amplex Red fluorescence as described (33). Following FFA measurement, the remaining samples were analyzed by LC-MS as described below.

### Phospholipid substrate specificity assay

To assess the phospholipid substrate specificity of mitochondrial ABHD4-2, phospholipid vesicles containing DOPC, phosphatidylglycerol (PG), CL, and phosphatidic acid (PA) (2:2:1:1, molar ratio) were prepared as described above. Cardiolipin-free control vesicles containing DOPC, PG, and PA (2:2:1, molar ratio) were prepared in parallel. Vesicles were incubated with 2 μg recombinant ABHD4-2 WT or the catalytic mutant ABHD4-2 S122A for 2.5 h, with no-enzyme controls included alongside these reactions. Reactions were terminated by the addition of chloroform/methanol (2:1, v/v), and lipids were extracted by the Bligh and Dyer method (34), dried, and analyzed by two-dimensional TLC. The first dimension was developed with chloroform/methanol/28% ammonium hydroxide (65:25:4, v/v/v), followed by the second dimension with chloroform/acetone/methanol/acetic acid/water (50:20:10:10:5, v/v/v/v/v). TLC plates were air-dried, sprayed with 10% (w/v) cupric sulfate in 8% (v/v) phosphoric acid, and charred at 180°C for 10 min. Lipid spots were quantified by densitometry using Image Lab software (Bio-Rad). FFA values were scaled by 10^6^ and reported as arbitrary units (a.u.). Background-subtracted band intensities were used for quantification, and the remaining substrate for each phospholipid was normalized to the corresponding no-enzyme control, defined as 100%.

### Lipid extraction and LC-MS analysis

Lipids were extracted at 4°C using methanol:chloroform (2:1, v/v) containing CL 14:0/14:0/14:0/14:0 as internal standard (400 pmol/sample). Briefly, 100 μl homogenate (60 μl assay sample plus 40 μl water) was mixed with 810 μl extraction solvent, incubated for 30 min on a rocking platform at 4°C, and centrifuged at 2,000g for 30 min. The pellet was re-extracted with 100 μl water and 400 μl chloroform:methanol (1:2, v/v), and supernatants were combined. Phase separation was induced by addition of 260 μl water, followed by centrifugation at 2,000g for 30 min. The upper aqueous phase was collected, and a second extraction of the lower phase was performed with 100 μl 0.01 M KCl. Combined aqueous fractions were adjusted to 2.0 ml with 50% methanol, lyophilized, and stored for further analysis. The lower organic phase was dried under nitrogen and stored at −20°C until lipidomic analysis.

Dried extracts were resuspended in 50 μl solvent A, centrifuged at 3000 rpm for 3 min, and 5 μl was injected for LC-MS analysis. Samples were randomized and analyzed in positive and negative electrospray ionization modes. Chromatographic separation was performed on an Agilent 1290 Infinity HPLC system equipped with a Zorbax Eclipse Plus C18 RRHD column (2.1 × 50 mm, 1.8 μm) and guard column at 45°C. The autosampler was maintained at 8°C. Solvent A consisted of isopropanol:methanol:water (50:10:40, v/v/v) and solvent B of isopropanol:water (99:1, v/v), both containing 5 mm ammonium acetate and 0.1% acetic acid. The flow rate was 0.35 ml/min, and the gradient was as follows: 0–3 min, 100% A; 3–5 min, 20% B; 5–25 min, 30% B; 25–35 min, 95% B; 35–36 min, 95% B; 36–38 min, 100% A, followed by 2 min re-equilibration. Mass detection was performed on an Agilent 6530 Q-TOF instrument over an m/z range of 70–1700. Lipid identification was supported by pooled quality-control samples analyzed in all-ion fragmentation mode at collision energies of 10, 20, and 40 V.

### TLC analysis of CL hydrolysis

Cardiolipin hydrolysis by ABHD4 was measured using fluorescent (TopFluor TMR-cardiolipin, 5 μm) or native bovine heart cardiolipin (50 μm). Reactions were performed in buffer containing 50 mm Tris-HCl (pH 8.0), 3 mm MgCl_2_, 1 mm EDTA, 5 mm CaCl_2_, 250 μm Chaps, and 40% glycerol, as described previously (35). Reactions (250 μl) were initiated by adding purified ABHD4 (1 μg) and incubated at 37°C. TMR-cardiolipin assays were performed for 15 min, whereas native cardiolipin reactions were incubated for 1 h unless otherwise indicated. For protein dependence, 0.25–4 μg enzyme was used for 1 h. For time-course assays, 1 μg enzyme was incubated for 0.5–3 h. Catalytic mutants (S159A and S122A) were included where indicated. Reactions were stopped by lipid extraction using the Bligh and Dyer method (34). Lipids were separated by TLC using chloroform:methanol:water (65:25:4, v/v/v). Lipid standards were run in parallel for identification. Fluorescent lipids were detected using an Azure Biosystems c600 imaging system. The lipids were visualized by iodine vapor or by charring after staining with 10% (w/v) cupric sulfate in 8% (v/v) phosphoric acid. Band intensities were quantified using ImageJ (36), and enzymatic activity was determined by monitoring cardiolipin depletion and FFA formation.

### Mitochondrial model bilayer preparation

A synthetic lipid bilayer was generated in vitro to mimic the lipid composition of rat liver mitochondria, as described previously (37, 38). Lipid substrates were mixed in chloroform at the following molar ratios: 40% DOPC, 34% DOPE, 18% tetralinoleoyl cardiolipin (TOCL), 5% phosphatidylinositol (PI), and 3% DOPS. The lipid mixture was dried under a stream of N_2_ to form a thin film and subsequently rehydrated in assay buffer containing 50 mm Hepes (pH 7.4), 80 mm KCl, 3 mm MgCl_2_, 2 mm CaCl_2_, and 1 mm DTT. The suspension was vortexed to generate multilamellar phospholipid vesicles, which were used for enzymatic assays. Purified ABHD4-2 (1 μg) was incubated with the vesicles at 37°C for 30 min in a total reaction volume of 250 μl. For time-course experiments, the enzyme was incubated for 0.5–2.5 h under the same conditions. Reactions were stopped by addition of chloroform:methanol (2:1, v/v), followed by lipid extraction and separation by TLC using chloroform:methanol:water (65:25:4, v/v/v). Lipid hydrolysis was evaluated by monitoring depletion of CL and the corresponding formation of FFA.

### Structural clustering and Foldseek analysis

Structural clustering and homolog identification of proteins were performed using Foldseek (v4.x) via the Foldseek web interface (39, 40, 41). The human α/β hydrolase domain-containing protein 5 (ABHD5) protein (UniProt: Q8WTS1), a close paralog of ABHD4, was used as the query for structure-based searches (42). The AlphaFold-predicted structure was obtained from the AlphaFold Protein Structure Database (43, 44) and used as input for Foldseek analysis with default parameters. Foldseek performs structure-based similarity searches using a 3Di structural alphabet, which represents local three-dimensional residue-residue interactions, and uses sequence alignment-like scoring enabling rapid comparison of three-dimensional protein structures across large scale datasets to identify structurally related proteins. Cluster information, representative structures, and taxonomic distribution were obtained from the Foldseek output. Structural confidence scores and domain organization were based on AlphaFold annotations. Structural clustering and evolutionary relationships were visualized using the Foldseek web interface and further refined for presentation. Foldseek was employed for structure-based alignment and clustering of structurally similar protein structures.

### Sequence analysis, structural modeling, and docking

Protein sequences for murine ABHD4, ABHD5, yeast Cld1p, and *Drosophila* Pummelig were retrieved from the NCBI GenBank (45). Homologs were identified based on sequence similarity and conserved domain architecture. Exon-intron organization was obtained from Ensembl (46). AlphaFold-predicted structures were obtained from the AlphaFold Protein Structure Database (v4) (43, 44). The three-dimensional structures of mouse ABHD4-1 (UniProt: Q3U7M5), ABHD4-2 (Q8C0M2), yeast Cld1p (P53264), Pummelig-1 (Q5U191), Pummelig-2 (Q7JQU9), and human ABHD5 (Q8WTS1) were used for structural comparisons. Structural superimposition and pairwise Cα RMSD calculations were performed in PyMOL (v2.5) using the super command with default parameters. Alignments were based on backbone atoms of the conserved core β-sheet, consisting of eight β-strands connected by α-helices. The catalytic triad (Ser-Asp-His) was analyzed across all structures, with residues visualized in stick representation to assess spatial conservation. Molecular docking was performed using AutoDock Vina (v1.2) (47) and SwissDock with default parameters (48). A representative CL substrate, tetralinoleoyl cardiolipin, was used for docking. In ABHD4, the catalytic triad (Ser, Asp, and His) was conserved, consistent with the GXSXG motif characteristic of serine hydrolases.

### Generation of doxycycline-inducible ABHD4-2 stable cell lines

Full-length ABHD4-2 (WT) and the catalytic mutant ABHD4-2 (S122A) were fused in frame at the C terminus to enhanced cyan fluorescent protein (ECFP) and cloned under a TRE promoter in a Tet-On lentiviral backbone (pLVX-TetOne-Puro or pInducer). All constructs were sequence verified prior to use. Lentivirus was produced in HEK293T cells using psPAX2 and pMD2.G, collected at 48–72 h, filtered (0.45 μm), and used immediately for transduction. BAs were transduced at a multiplicity of infection of 0.3–1 in the presence of 8 μg/ml polybrene for 16–24 h, followed by a 24 h recovery. Cells were then selected with puromycin (2–3 μg/ml) or G418 (500–800 μg/ml) for 5–7 days until nontransduced controls were fully eliminated. When needed, single-cell clones were isolated by limiting dilution to obtain uniform expression, otherwise, pooled populations were maintained. Expression was induced with 1 μg/ml doxycycline for 36–48 h in complete phenol red–free medium. To ensure comparable expression between WT and S122A, doxycycline titration (0–1000 ng/ml) was performed, and conditions were selected based on matched ECFP signal with minimal background in uninduced cells. Expression was routinely confirmed by live-cell ECFP fluorescence or immunoblotting using anti-GFP/CFP and anti-ABHD4 antibodies. Confocal imaging was used to verify mitochondrial localization of the expressed constructs. Cell lines showing detectable basal expression in the absence of doxycycline were excluded or re-cloned. All cultures were regularly tested for mycoplasma contamination, and experiments were performed between passages 3 and 15 postselection. Independent transductions yielded consistent expression and localization patterns in mitochondria.

### JC-1 analysis (Δψm)

Mitochondrial membrane potential (Δψm) was assessed using the ratiometric dye JC-1 (Thermo Fisher, Cat. No. T3168) (49, 50). BAs expressing ABHD4-WT or S122A were plated on 25 mm glass coverslips prior to imaging. Cells were washed gently and incubated with JC-1 (2 μM) in phenol red-free DMEM for 30 min at 37°C, washed with prewarmed imaging buffer, and imaged immediately. Live-cell imaging was performed using an Andor Dragonfly spinning-disk confocal microscope equipped with a 63 × 1.4 NA oil immersion objective. ECFP, JC-1 green (monomers), and JC-1 red (aggregates) signals were acquired using 445 nm, 514 nm, and 561 nm excitation, respectively, with identical acquisition settings across conditions. Cells were defined using whole-cell regions of interest in ImageJ (Fiji) (36), and fluorescence signals were quantified within these regions. Quantification was performed using a custom Python script to extract JC-1 red and green intensities and calculate per-cell red/green ratios. For ABHD4-WT and S122A conditions, ECFP-positive cells were analyzed. Data were processed per batch and combined for final analysis. Carbonyl cyanide 4-(trifluoromethoxy)phenylhydrazone (FCCP) (10 μm, 30 min) and rotenone (1 μm, 30 min) were included as depolarization controls. A decrease in the JC-1 red/green ratio was interpreted as mitochondrial depolarization. Reciprocal green/red ratios were calculated as a validation and yielded consistent results. Statistical significance was assessed using two-sided Mann-Whitney tests. All analysis parameters were applied consistently across conditions within each experiment.

### Mitotracker deep red staining

Cells were incubated with MitoTracker™ Deep Red FM (50 nM; Thermo Fisher, Cat. No. M22426) for 30 min at 37°C, washed with prewarmed Krebs buffer, and imaged immediately. ECFP and MitoTracker signals were acquired using 445 nm and 637 nm excitation, respectively, with identical acquisition settings across conditions. FCCP (10 μm, 30 min) and rotenone (1 μm, 30 min) were included as depolarization controls. Mitochondria were segmented from the MitoTracker channel, and segmentation quality was verified prior to analysis. Fluorescence intensities were quantified on a per-cell basis.

### Statistics

Statistical analyses were performed using methods appropriate for each dataset. All experiments were conducted with at least three independent biological replicates. Biochemical data are presented as mean ± SD and were analyzed using two-sided Student’s *t* test. Single-cell imaging data (JC-1 and MitoTracker) are shown as median values with individual data points and were analyzed using the Mann-Whitney U test. For imaging experiments, the sample size (N) represents individual cells pooled from three independent experiments, whereas for biochemical assays, the sample size (N) refers to biological replicates. Statistical significance is indicated as ∗*P* < 0.05, ∗∗*P* < 0.01, ∗∗∗*P* < 0.001, and ∗∗∗∗*P* < 0.0001.

## Results

### ABHD4/5, Cld1p, and Pummelig are evolutionarily conserved structural paralogs

**Fig. 1:**
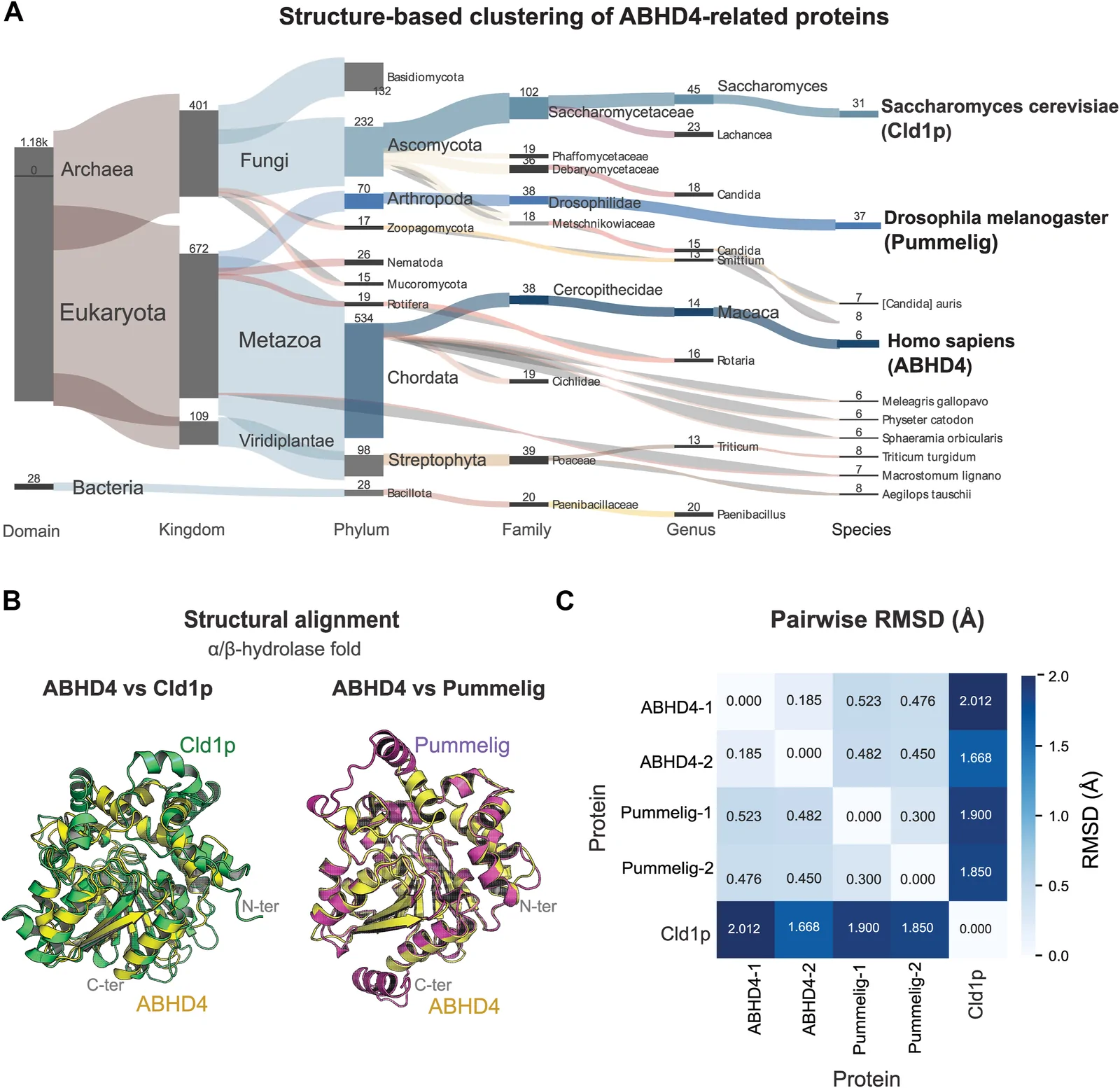
ABHD4, Cld1p, and Pummelig are evolutionarily conserved structural paralogs. A: Structure-based clustering of ABHD4-related homologs across major taxonomic groups using Foldseek analysis (39, 40, 41). Branch width reflects the relative number of sequences assigned to each lineage. Colored branches highlight representative homologs from yeast (*Saccharomyces cerevisiae*, Cld1p), fly (*Drosophila melanogaster*, Pummelig), and human (ABHD4), whereas remaining branches are shown in gray. B: Superposition of ABHD4 (yellow) with Cld1p (green) and Pummelig (magenta) shows conservation of the α/β-hydrolase fold in evolution. N and C termini are indicated. C: Pairwise structural similarity among ABHD4 isoforms, Pummelig isoforms, and Cld1p based on AlphaFold models. Heatmap values represent Cα RMSD (Å) derived from structural alignments, with lower values indicating greater structural similarity.

Structure-based protein clustering using Foldseek (39, 40, 41) indicates that ABHD4 belongs to a conserved α/β-hydrolase family (51) whose members are distributed across fungi, plants, insects, and mammals, indicating divergence from a common ancestral enzyme >500 million years ago (52) (Fig. 1A).

Sequence and structure-based analyses identify ABHD4 as the closest mammalian homolog of the yeast CL lipase Cld1p (13) and the *Drosophila* protein Pummelig (53). BLAST analysis supports this relationship, showing 26% identity (42% conservation) with Cld1p and higher similarity toward Pummelig (49% identity, 68% conservation), consistent with a shared evolutionary origin.

Structural comparisons using AlphaFold predicted models further reinforce this conservation. Comparison of ABHD4-1 and ABHD4-2 showed that the two isoforms are nearly superimposable (Cα RMSD = 0.185 Å), differing only in the N-terminal region, which is absent in ABHD4-2. The α/β-hydrolase core remains conserved, indicating that N-terminal truncation does not alter the overall protein fold (supplemental Fig. S1B). Across these taxa, the α/β-hydrolase fold is preserved despite substantial sequence divergence, with most variation localized to the N-terminal region, likely reflecting lineage-specific targeting or regulatory adaptations. ABHD4 isoforms exhibit strong structural similarity to Pummelig (RMSD = 0.523 Å for ABHD4-1 and 0.476 Å for ABHD4-2) and align with Cld1p (RMSD = 1.668 2.012 Å) (Fig. 1B, C and supplemental Fig. S1).

Within mammals, ABHD5 is the closest paralog of ABHD4 that shares this overall structural framework but lacks the catalytic serine nucleophile (S155N substitution) and functions as a nonenzymatic coactivator of PNPLA2 (and other PNPLA paralogs) rather than as a hydrolase (54, 55, 56).

Together, these data indicate that ABHD4, Cld1p, and Pummelig represent conserved enzymatic members of this α/β-hydrolase lineage, whereas ABHD5 is a structurally related pseudoenzyme that has diverged functionally following gene duplication.

### ABHD4-2 is highly expressed in mitochondrial rich tissues

**Fig. 2:**
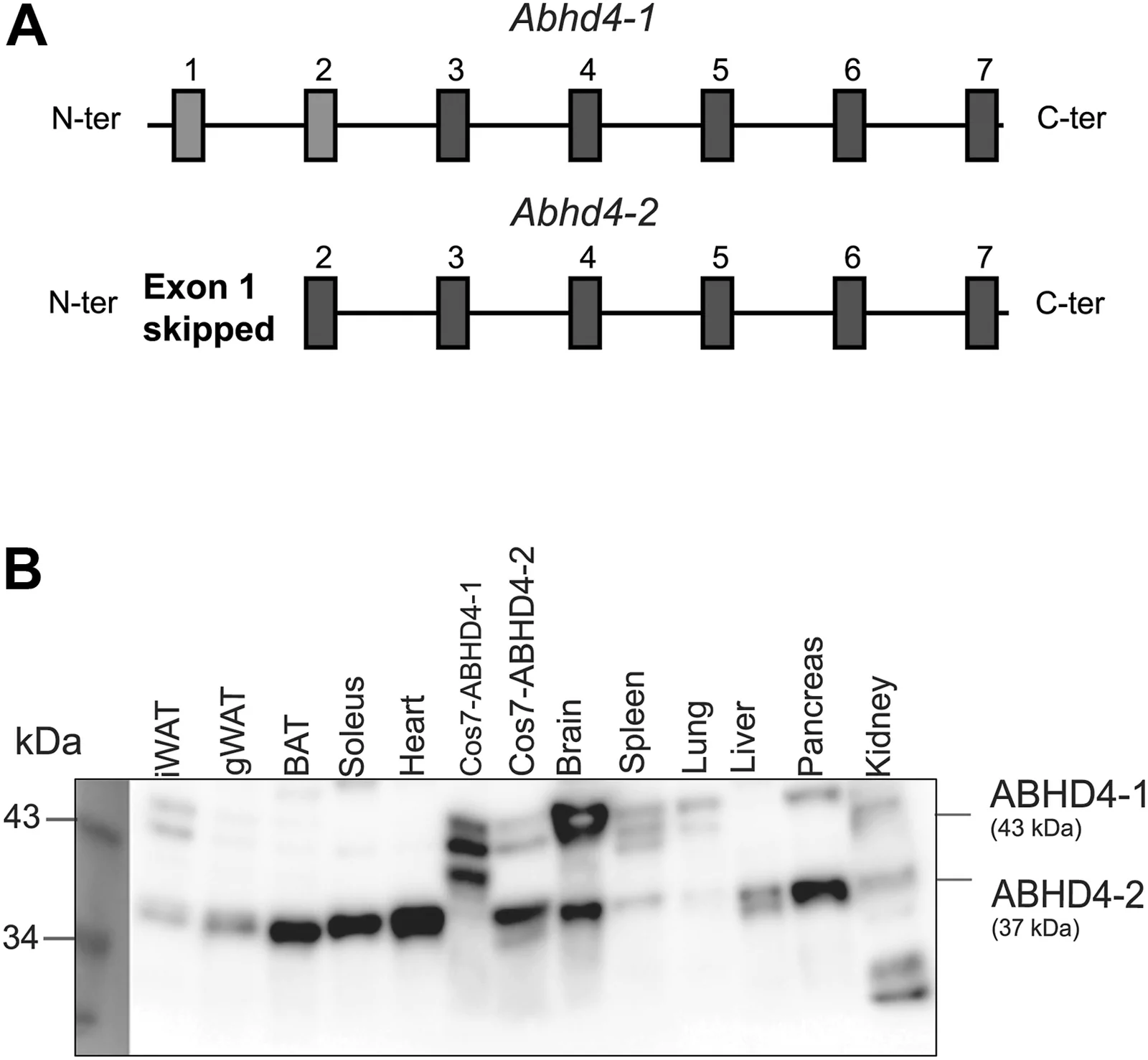
Isoform-specific organization and tissue distribution of ABHD4. A: Schematic representation of murine *Abhd4* gene isoforms generated by alternative splicing. ABHD4-1 contains exons 1–7, whereas ABHD4-2 lacks exon 1, resulting in an N-terminal truncation. B: Tissue distribution of murine ABHD4 isoforms determined by immunoblotting. ABHD4-1 and ABHD4-2 were detected using an anti-ABHD4 antibody, and apparent molecular weights are indicated.

Analysis of *Abhd4* gene and gene product sequences using GenBank reveals that the mammalian *Abhd4* gene produces two alternative splice variants: a longer isoform (ABHD4-1) and a shorter isoform (ABHD4-2). Exon mapping showed that ABHD4-1 contains exons 1–7, whereas ABHD4-2 lacks exon 1 and initiates within exon 2, resulting in an N-terminal truncation (Fig. 2A). Interestingly, this general intron-exon structure is conserved in ABHD4, ABHD5, and Pummelig (53).

To assess the tissue distribution of ABHD4 isoforms in vivo, we performed immunoblot analysis of protein lysates from multiple mouse organs. Both isoforms were detected across several tissues, including soleus muscle, heart, brain, spleen, lung, liver, pancreas, and kidney (Fig. 2B). ABHD4-1 migrated at ∼43 kDa, whereas ABHD4-2 migrated at ∼37–38 kDa, consistent with their predicted molecular weights. Notably, ABHD4-2 was most abundant in BAT, heart, and soleus muscle, tissues with high mitochondrial content and oxidative capacity. In BAT, subcellular fractionation showed enrichment of ABHD4-2 in mitochondrial fractions (supplemental Fig. S2). The enrichment of ABHD4-2 in oxidative tissues and mitochondrial fractions supports a role for ABHD4-2 in mitochondrial lipid metabolism.

### ABHD4 and Pummelig isoforms exhibit distinct subcellular localization

**Fig. 3:**
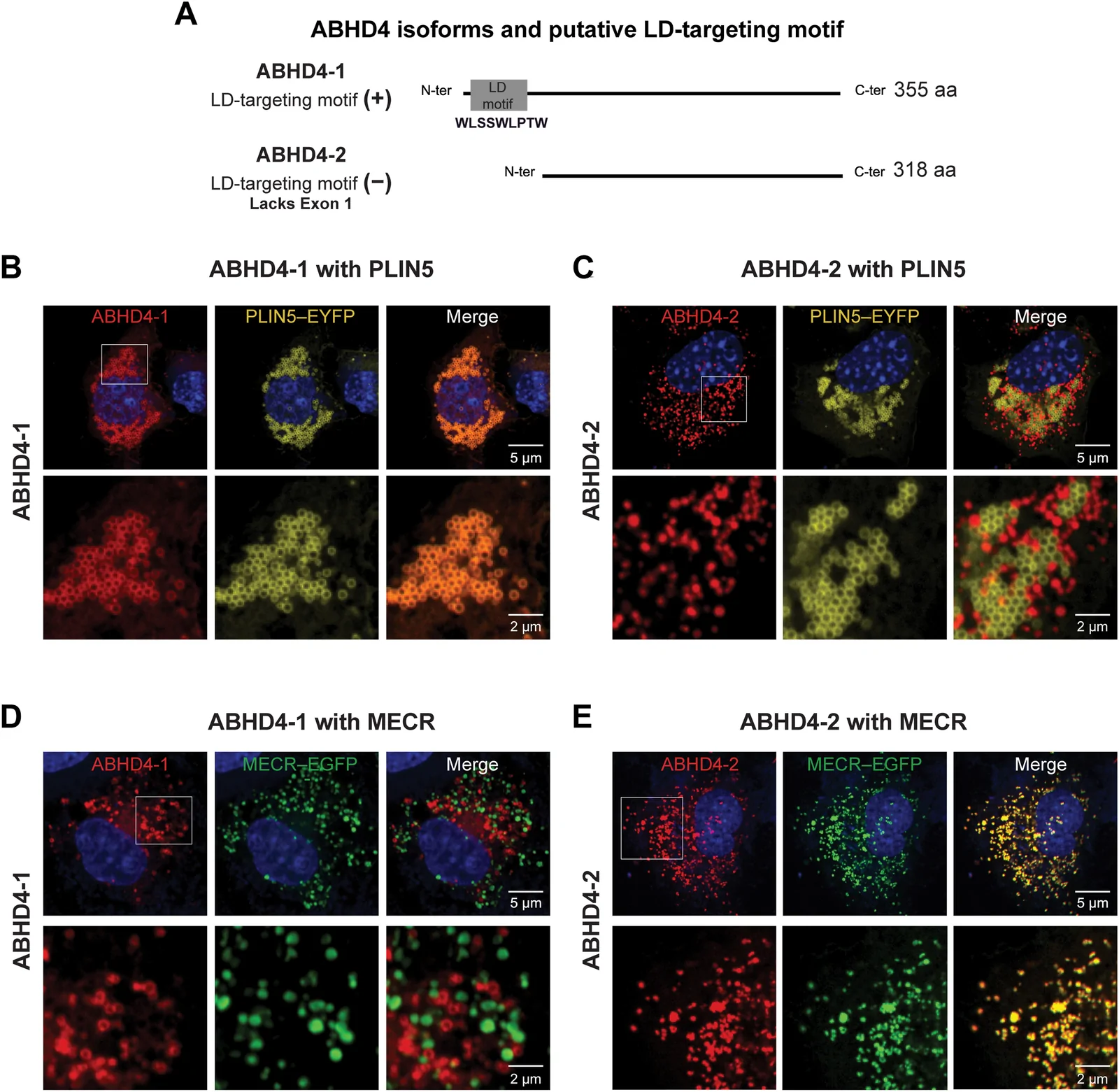
Isoform-specific subcellular localization of ABHD4 in COS-7 cells. Confocal imaging of COS-7 cells cotransfected with fluorescently tagged ABHD4 isoforms and organelle-specific markers. Cells were incubated overnight with 200 μm oleic acid (16–20 h) prior to imaging. ABHD4-1 (long isoform) and ABHD4-2 (short isoform) show distinct subcellular distributions. A: Domain organization of ABHD4 isoforms. The long isoform, ABHD4-1 (355 aa), contains an N-terminal region with a putative lipid droplet (LD)-targeting motif, whereas the short isoform, ABHD4-2 (318 aa), lacks this region due to exon 1 skipping. B and C: ABHD4 isoforms and lipid droplets. COS-7 cells expressing mCherry-tagged ABHD4 constructs (red) were coexpressed with PLIN5-EYFP (Perilipin 5; LD marker). ABHD4-1 is observed in association with PLIN5-positive LD structures, consistent with the presence of the N-terminal LD-targeting motif, whereas ABHD4-2 shows minimal overlap under the same conditions. D and E: ABHD4 isoforms and mitochondria. COS-7 cells expressing mCherry-tagged ABHD4 constructs (red) were coexpressed with MECR-EGFP (mitochondrial trans-2-enoyl-CoA reductase; mitochondrial marker). ABHD4-2 is observed in mitochondrial regions, whereas ABHD4-1 shows comparatively limited overlap, consistent with isoform-dependent mitochondrial association. Lower panels show magnified views of the corresponding regions above. Scale bars, 5 μm (top panels) and 2 μm (zoom panels). A similar isoform-dependent localization pattern was observed for Pummelig-1 and Pummelig-2, indicating a conserved distribution across these homologs (see supplemental Fig. S3)_._ MECR, mitochondrial trans-2-enoyl-CoA reductase.

The splice variants of ABHD4 and Pummelig differ in the presence or absence of an N-terminal extension. As reported for ABHD5 (57), this N-terminal region contains a lipid droplet (LD)-targeting motif, which is present only in the longer isoforms, ABHD4-1 and Pummelig-1, suggesting conserved isoform-specific targeting (Fig. 3A). To test whether this feature determines subcellular localization, we expressed fluorescently tagged ABHD4 and Pummelig isoforms in COS-7 cells together with organelle-specific markers. Consistent with this prediction, ABHD4-1 targeted LDs and colocalized with Perilipin 5-EYFP (Perilipin 5, a LD marker) (Fig. 3B). In contrast, the shorter isoform, ABHD4-2, showed no detectable colocalization with Perilipin 5 (Fig. 3C) and instead localized to mitochondria, as indicated by colocalization with mitochondrial trans-2-enoyl-CoA reductase-EGFP (a mitochondrial marker) (58) (Fig. 3D, E). Notably, Pummelig isoforms exhibited a similar pattern, with Pummelig-1 localizing to LDs and Pummelig-2 associating with mitochondria (supplemental Fig. S3), indicating conserved isoform-specific subcellular distribution. In addition, these observations were further supported by LipidTOX Deep Red staining (supplemental Fig. S4). Together, the confocal imaging data demonstrate that ABHD4-1 and Pummelig-1 localize to LDs, whereas ABHD4-2 and Pummelig-2 are targeted to mitochondria.

### ABHD4-2 and Pummelig-2 localize to the cytosolic face of the OMM

**Fig. 4:**
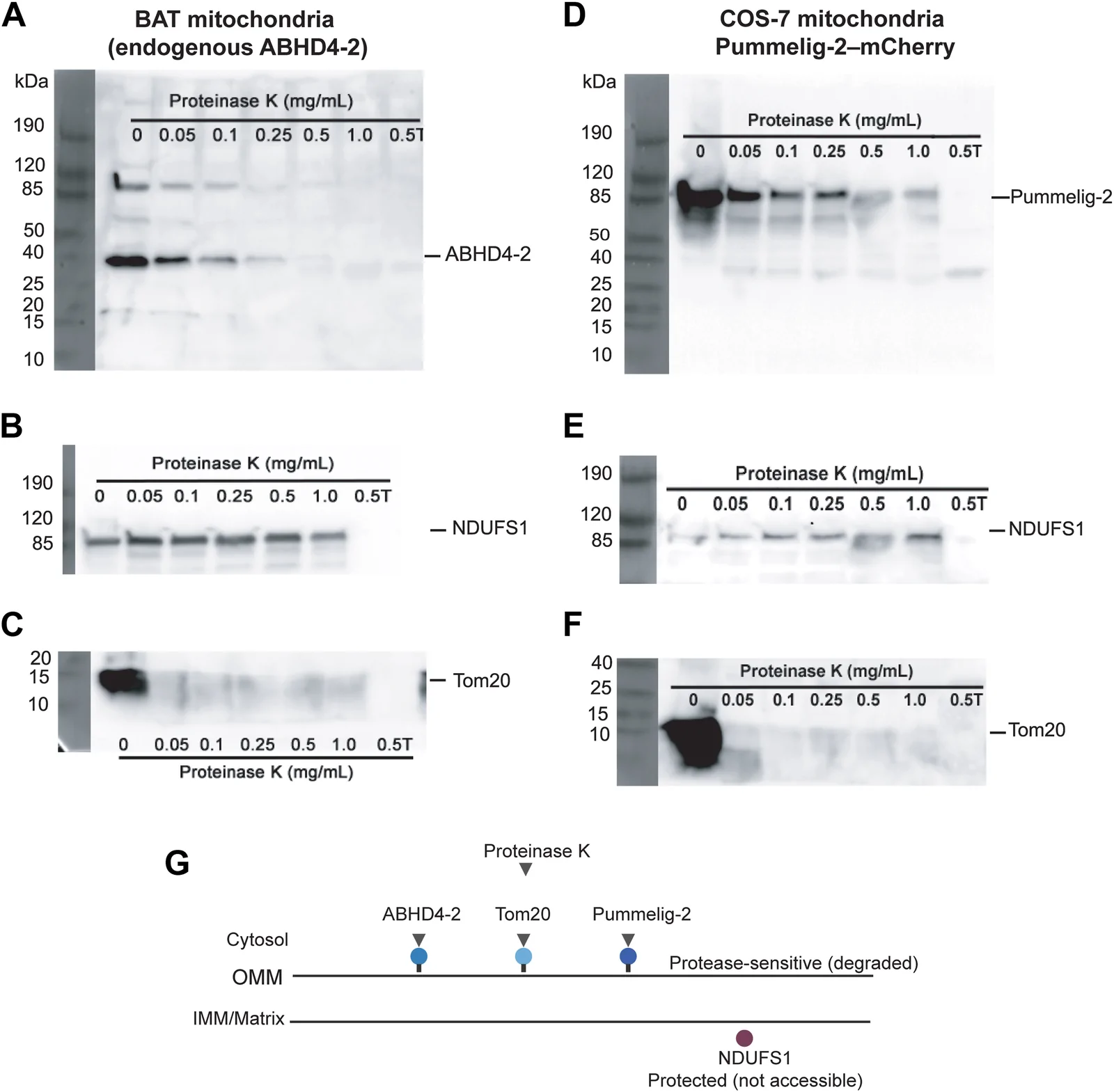
Endogenous ABHD4-2 and Pummelig-2 are accessible to proteinase K at the outer mitochondrial membrane. Proteinase K protection assays were performed on mitochondria isolated from mouse brown adipose tissue (BAT) or COS-7 cells. Mitochondria were treated with increasing concentrations of proteinase K (0–1 mg/ml) for 15 min at 37°C in the absence or presence of Triton X-100 (T, 0.5%). A–C: Endogenous ABHD4-2 in BAT mitochondria shows a concentration-dependent loss of signal upon proteinase K treatment (A), similar to the outer mitochondrial membrane marker TOM20 (C). The inner membrane/matrix protein NDUFS1 remains protected under the same conditions (B). D–F: In COS-7 cells expressing Pummelig-2–mCherry, Pummelig-2 is sensitive to proteinase K treatment (D), comparable to TOM20 (F). NDUFS1 remains protected under identical conditions (E). G: Schematic model of submitochondrial accessibility. ABHD4-2 and Pummelig-2 are accessible to proteinase K, consistent with localization at the cytosolic face of the outer mitochondrial membrane. NDUFS1 is protected within the inner membrane/matrix compartment. Data are representative of three independent experiments.

Our results show that ABHD4-2 and Cld1p are closely related structural homologs targeted to mitochondria. In yeast, Cld1p is targeted to the IMM where it deacylates CL for acyl chain remodeling by tafazzin (59). Recent work indicates that ABHD18 performs a similar function in mammals (17). We therefore examined the submitochondrial localization of ABHD4-2 and Pummelig-2 in mitochondria isolated from BAT using proteinase K sensitivity assays. Under these conditions, proteins exposed to the OMM are sensitive to proteolysis, whereas IMM proteins, such as NDUFS1, remain protected. We observed that endogenous ABHD4-2 was progressively degraded with increasing concentrations of proteinase K (Fig. 4A), like the OMM marker TOM20 (Fig. 4C). In contrast, the IMM protein NDUFS1 remained fully protected under the same conditions (Fig. 4B). Similarly, mitochondria isolated from COS-7 cells overexpressing mCherry-tagged Pummelig-2 (PumB-mCherry) showed proteinase K-dependent degradation (Fig. 4D), comparable to TOM20 (Fig. 4F), whereas the IMM marker NDUFS1 remained protected (Fig. 4E). We further examined the submitochondrial localization of ABHD4-2 using additional proteinase K protection assays on mitochondria isolated from COS-7 cells expressing untagged ABHD4-2. ABHD4-2 was progressively degraded with increasing proteinase K, whereas cytochrome c, an IMS marker, remained protected in intact mitochondria and was degraded only after membrane permeabilization (supplemental Fig. S5), indicating that the IMS was inaccessible to proteinase K under intact conditions. ABHD4-2 is therefore exposed on the cytosolic surface of the OMM, rather than facing the IMS. Together, these results indicate that ABHD4-2 and Pummelig-2 localize to the cytosolic face of the OMM, distinguishing them from the IMM-localized CL hydrolase Cld1p.

### ABHD4 and Pummelig exhibit in vitro CL hydrolase activity: serine residues are essential for catalytic activity

To determine whether ABHD4 and Pummelig possess CL hydrolase activity, we expressed octa-histidine-tagged recombinant isoforms of both proteins in High Five insect cells using baculovirus infection and purified the proteins by cobalt affinity resin chromatography. Members of the ABHD family contain a conserved GXSXG motif, a hallmark of serine hydrolases (51, 52, 60). In ABHD4, this motif contains a catalytic serine residue (ABHD4-1: Ser159; ABHD4-2: Ser122) predicted to be required for CL hydrolysis. To test the functional importance of this residue and exclude the possibility of contaminant-mediated activity, we generated Ser→Ala catalytic mutants for both isoforms. Coomassie-stained SDS-PAGE confirmed comparable purity and loading of ABHD4-1 and ABHD4-2 wild-type and catalytic mutant proteins used in the biochemical assays (supplemental Fig. S6). Immunoblotting confirmed expression of both wild-type and mutant constructs for ABHD4 and Pummelig isoforms (supplemental Fig. S7).

**Fig. 5:**
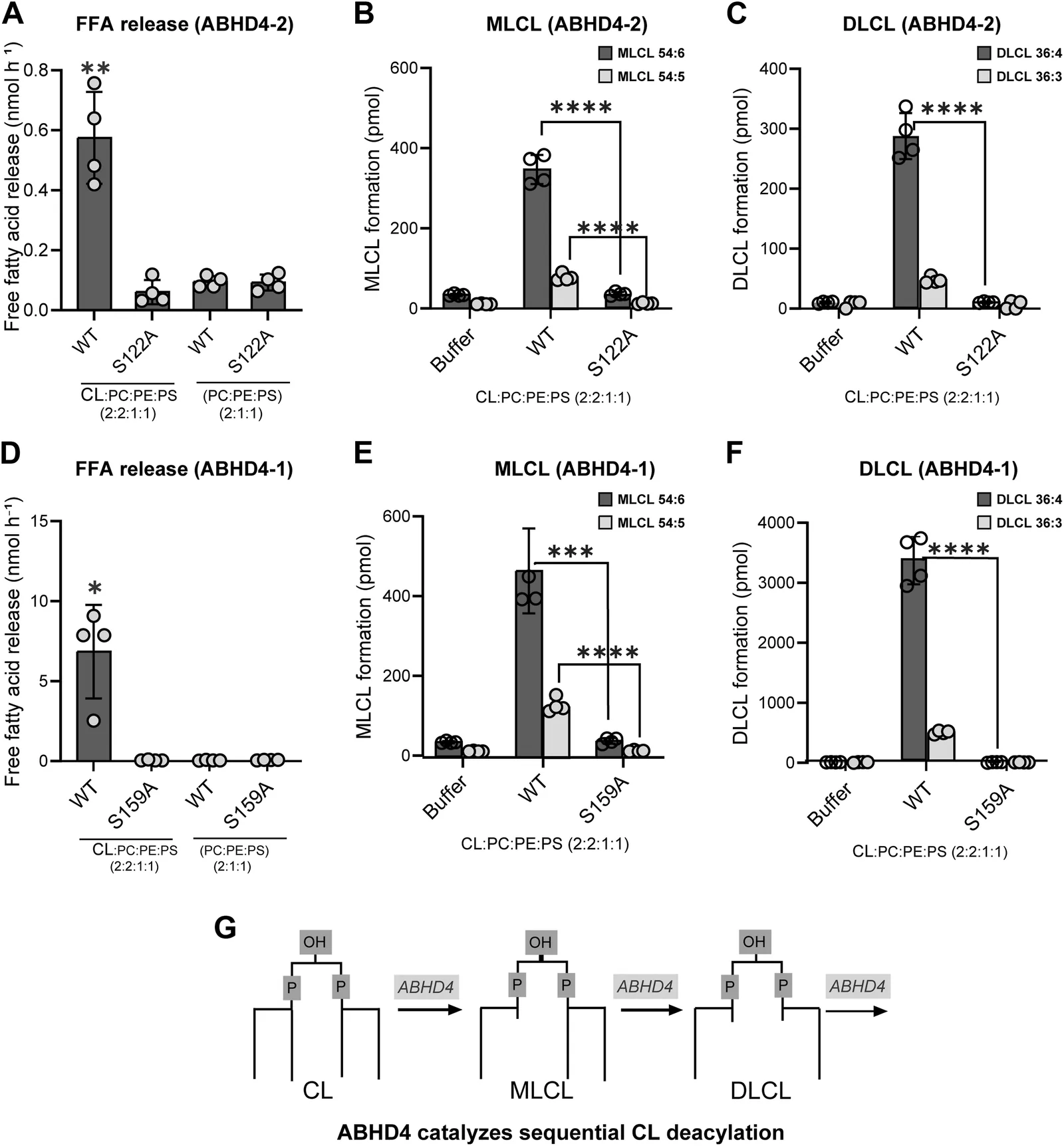
ABHD4 isoforms catalyze cardiolipin deacylation to generate MLCL and DLCL. ABHD4 isoforms catalyze CL hydrolysis, resulting in the sequential formation of monolysocardiolipin (MLCL) and dilysocardiolipin (DLCL) in vitro. Lipase assays were performed using mixed phospholipid vesicles containing CL (CL:PC:PE:PS, 2:2:1:1). Free fatty acid (FFA) release was measured, and lipid species were quantified by LC-MS, with CL (14:0/14:0/14:0/14:0) used as an internal standard. (A-C) ABHD4-2 CL lipase activity. A: FFA release increased with WT enzyme in the presence of CL, whereas the catalytic mutant S122A showed minimal activity. B: LC-MS quantification of MLCL species (54:6 and 54:5) showed increased product formation with WT ABHD4-2. C: DLCL species (36:4 and 36:3) were also increased. Product formation was reduced in the S122A mutant, consistent with dependence on the catalytic serine (Ser122). (D-F) ABHD4-1 activity under similar conditions. D: WT ABHD4-1 increased FFA release, whereas the S159A mutant showed minimal activity. (E) MLCL and (F) DLCL formation were detected with WT enzyme and reduced in the S159A mutant, consistent with dependence on the catalytic serine (Ser159). G: Schematic model of ABHD4-mediated CL deacylation. ABHD4 catalyzes stepwise removal of acyl chains from CL to generate MLCL and DLCL. Data represent independent reactions with individual data points shown and bars indicating mean values. Statistical significance is indicated as ∗*P* < 0.05, ∗∗*P* < 0.01, ∗∗∗*P* < 0.001, ∗∗∗∗*P* < 0.0001.

In vitro lipase activity was assessed using mixed phospholipid vesicles containing cardiolipin (+CL; CL:PC:PE:PS, 2:2:1:1) or lacking cardiolipin (−CL; PC:PE:PS, 2:1:1). FFA release by ABHD4-2 was observed predominantly in +CL vesicles, whereas −CL vesicles showed little, if any, FFA release (Fig. 5A). We next examined ABHD4-1 under the same conditions. Under + CL conditions, ABHD4-1 also exhibited robust FFA release, whereas the catalytic mutant S159A showed essentially no activity. Wild-type ABHD4-1 released virtually no FFA under −CL conditions (Fig. 5D).

Consistent with FFA formation, LC-MS/MS analysis demonstrated the formation of MLCL species (54:6 and 54:5) and DLCL species in reactions containing ABHD4-2, whereas the S122A mutant failed to generate these products (Fig. 5B, C). Under these conditions, ABHD4-1 also exhibited +CL-dependent hydrolysis, producing FFAs together with MLCL and DLCL species, whereas the catalytic mutant S159A did not have detectable activity (Fig. 5D-F). To determine whether this catalytic activity is conserved across homologous proteins, we next examined Pummelig. Recombinant Pummelig also showed robust CL hydrolase activity in the presence of CL, generating both MLCL and DLCL species, which was abolished in the S190A mutant (supplemental Fig. S8). Notably, reverting N155 to S155 in ABHD5 did not rescue CL hydrolase activity under these conditions (supplemental Fig. S9). These results support a conserved catalytic mechanism in ABHD4 and Pummelig toward CL that requires the active-site serine.

In addition, we performed a TLC-based CL hydrolysis assay using CL as the exclusive substrate under standard assay conditions. A protein dependence assay showed a dose-dependent increase in FFA release with a corresponding decrease in CL levels (0.25–4 μg enzyme) (supplemental Fig. S10A). A time course assay (supplemental Fig. S10B) demonstrated progressive CL degradation and confirmed sustained CL hydrolysis over time. A schematic model illustrating ABHD4-mediated stepwise CL deacylation is shown in Figure 5G. Collectively, biochemical analyses using recombinant proteins demonstrate that ABHD4 isoforms and Pummelig catalyze stepwise CL hydrolysis through their conserved active-site serine, resulting in the formation of FFAs, MLCL, and DLCL as metabolic products.

### ABHD4 isoforms and Pummelig exhibit phospholipase activity with differential CL-dependency

**Fig. 6:**
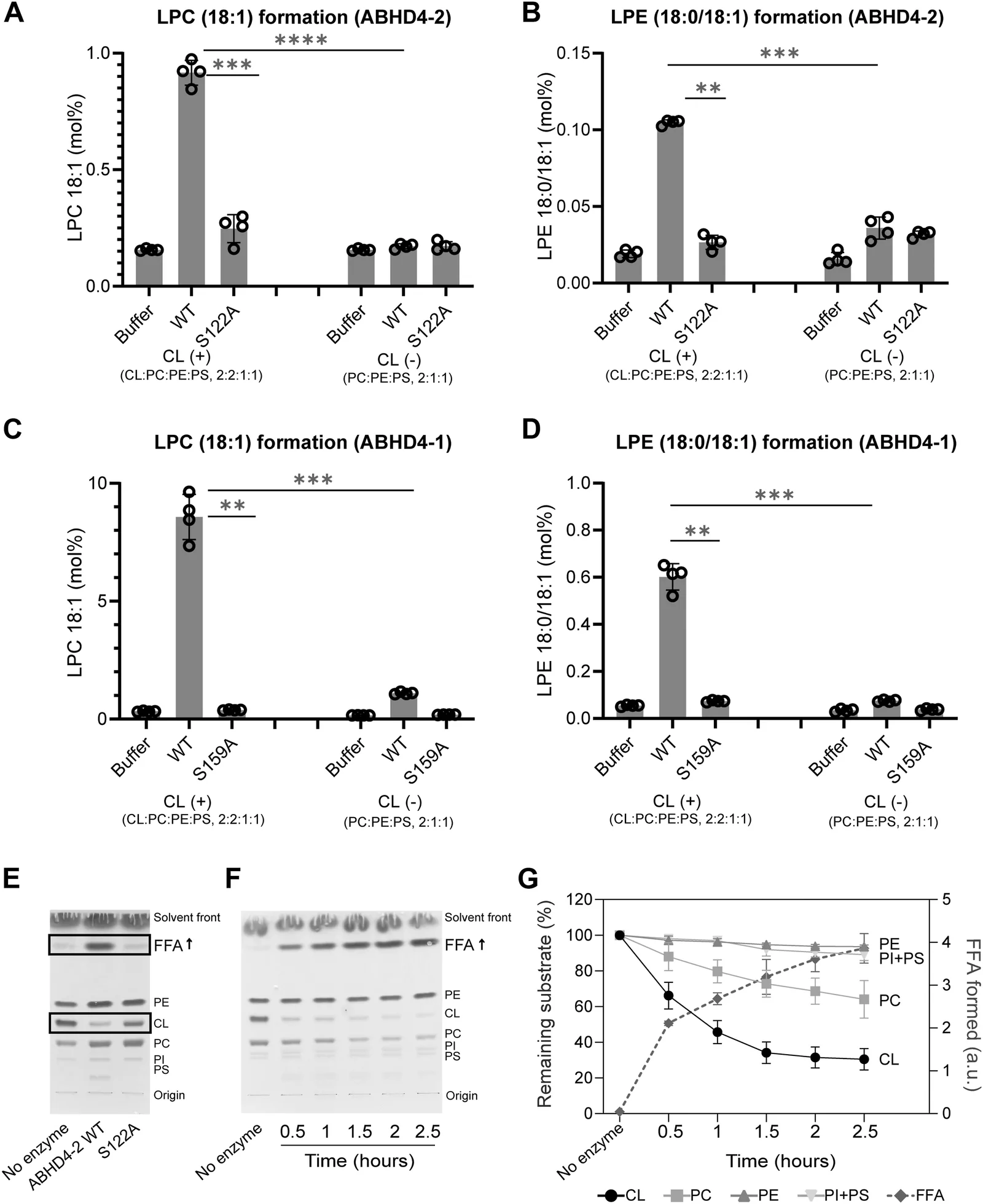
ABHD4 isoforms exhibit cardiolipin-dependent phospholipase activity with preferential CL hydrolysis in a mitochondrial model bilayer. A and B: Lysophospholipid formation was quantified by LC–MS following incubation of mixed phospholipid vesicles with ABHD4-2. Vesicles containing cardiolipin (+CL; CL:PC:PE:PS, 2:2:1:1) or lacking cardiolipin (−CL; PC:PE:PS, 2:1:1). A: LPC 18:1 formation was increased under +CL conditions with WT ABHD4-2. In the absence of CL, formation was reduced, and activity was minimal in the S122A mutant. B: LPE (18:0/18:1) formation showed a similar trend, with increased formation under + CL conditions, reduced formation in −CL vesicles, and decreased activity in the S122A mutant. C and D: Lysophospholipid formation by ABHD4-1 was measured under similar conditions. (C) LPC 18:1 and (D) LPE (18:0/18:1) were elevated in +CL vesicles with WT ABHD4-1. Activity was minimal in the S159A mutant, and product formation was reduced in −CL vesicles. These results indicate that ABHD4 activity toward PC and PE is enhanced by CL and depends on the catalytic serine. Data represent independent reactions with individual data points shown and bars indicating mean values. Statistical significance is indicated as ∗∗*P* < 0.01, ∗∗∗*P* < 0.001, ∗∗∗∗*P* < 0.0001. E: Representative TLC of a lipid bilayer approximating the phospholipid composition of rat liver mitochondria, incubated with ABHD4-2 WT or the catalytic mutant S122A for 30 min. Boxes mark the CL and FFA regions. F: Representative time-course TLC of the mitochondrial model bilayer incubated with ABHD4-2 WT for 0.5–2.5 h. G: Quantification of the TLC time course shown in panel F. Remaining phospholipid substrates were normalized to the no-enzyme control (100%) and plotted on the left y-axis. FFA formation is plotted on the right y-axis in arbitrary units. CL shows the greatest and most rapid reduction among the substrates tested, with the majority of hydrolysis occurring within the first 30 min; CL levels continue to decline, accompanied by a corresponding increase in FFA. PC also decreases, though to a lesser extent than CL, while PE and PI + PS remain largely unchanged. Data are mean ± SD (n = 3).

To determine whether CL modulates ABHD4 phospholipase activity toward other phospholipids, we quantified lysophospholipid formation by LC-MS analysis in mixed phospholipid vesicles under the same assay conditions, with or without CL (+CL: CL:PC:PE:PS, 2:2:1:1; −CL: PC:PE:PS, 2:1:1). ABHD4-2 significantly increased lysophosphatidylcholine (LPC) 18:1 formation in the presence of CL but not in the absence (Fig. 6A). As expected, LPC formation was abolished in the catalytic mutant S122A, indicating that LPC generation depends on catalytic activity and is enhanced in the presence of CL. Similarly, ABHD4-2 generated lysophosphatidylethanolamine (LPE) species only under + CL conditions (Fig. 6B). ABHD4-1 showed a similar pattern, with LPC and LPE species formation strongly promoted under + CL conditions (Fig. 6C, D). In contrast, while Pummelig also generated LPC and LPE species, this activity was independent of CL (supplemental Fig. S11).

### ABHD4-2 preferentially hydrolyzes CL over PG and PA

PG and PA are biosynthetic precursors of CL (5). We therefore examined the ability of ABHD4-2 to hydrolyze these lipids relative to CL. Vesicles contained PC, PG, CL, and PA (+CL; PC:PG:CL:PA), with PC included to promote stable vesicle formation. CL-free vesicles (−CL; PC:PG:PA) were analyzed under the same conditions. For comparison, we also analyzed the phospholipid composition used earlier for LC-MS (PC:PE:CL:PS) by two-dimensional TLC (supplemental Fig. S12A, B). Reaction products were identified by TLC visualization and quantified by densitometric analysis.

ABHD4-2 WT generated detectable FFA, lysocardiolipin, and LPC in CL-containing vesicles (supplemental Fig. S12A, B). No FFA or lysophospholipid products were detected in CL-free vesicles or in CL-containing vesicles incubated with the catalytic mutant S122A (supplemental Fig. S13A, B). ABHD4-2 hydrolyzed CL to the greatest extent in both vesicle compositions, whereas PG (PC:PG:CL:PA vesicles) and PC (PC:PE:CL:PS vesicles) were also hydrolyzed significantly, but less extensively than CL. PA and PE showed modest, nonsignificant decreases under the same conditions (supplemental Fig. S12C, D). Similarly, PG hydrolysis was observed only in the presence of CL, and LC-MS analysis showed that LPC and LPE formation (Fig. 6A, B) also required CL. Overall, ABHD4-2 preferentially hydrolyzes CL, and its broader activity toward PG and PC depends on the presence of CL within the bilayer.

### ABHD4-2 preferentially hydrolyzes CL in model mitochondrial lipid bilayers

To examine the substrate preference of ABHD4-2 under mitochondrial conditions, we constructed a heterogeneous lipid bilayer containing PC, PE, CL, PI, and PS at proportions approximating the reported lipid composition of rat liver mitochondria (37, 38). PG and PA were examined separately in independent liposome assays as described above (supplemental Figs. S12, S13). Incubation of these bilayers with ABHD4-2 reduced CL levels and increased FFA formation (Fig. 6E), supporting CL-directed lipase activity. In contrast, the catalytic mutant (S122A) showed minimal CL depletion and FFA generation. Time-course analysis further demonstrated that fatty acids generated by ABHD4-2 during the first 30 min could be attributed predominantly to the hydrolysis of CL (Fig. 6F, G). PC hydrolysis was modest during the first hour and increased with prolonged incubation, reaching approximately 64% of the initial input by 2.5 h, whereas PE and PI + PS were not significantly hydrolyzed by ABHD4-2 (Fig. 6G). Together, these results indicate that ABHD4-2 preferentially hydrolyzes CL in this mitochondrial lipid bilayer model.

### A conserved catalytic triad supports CL lipase activity in ABHD4, Pummelig, and Cld1p

**Fig. 7:**
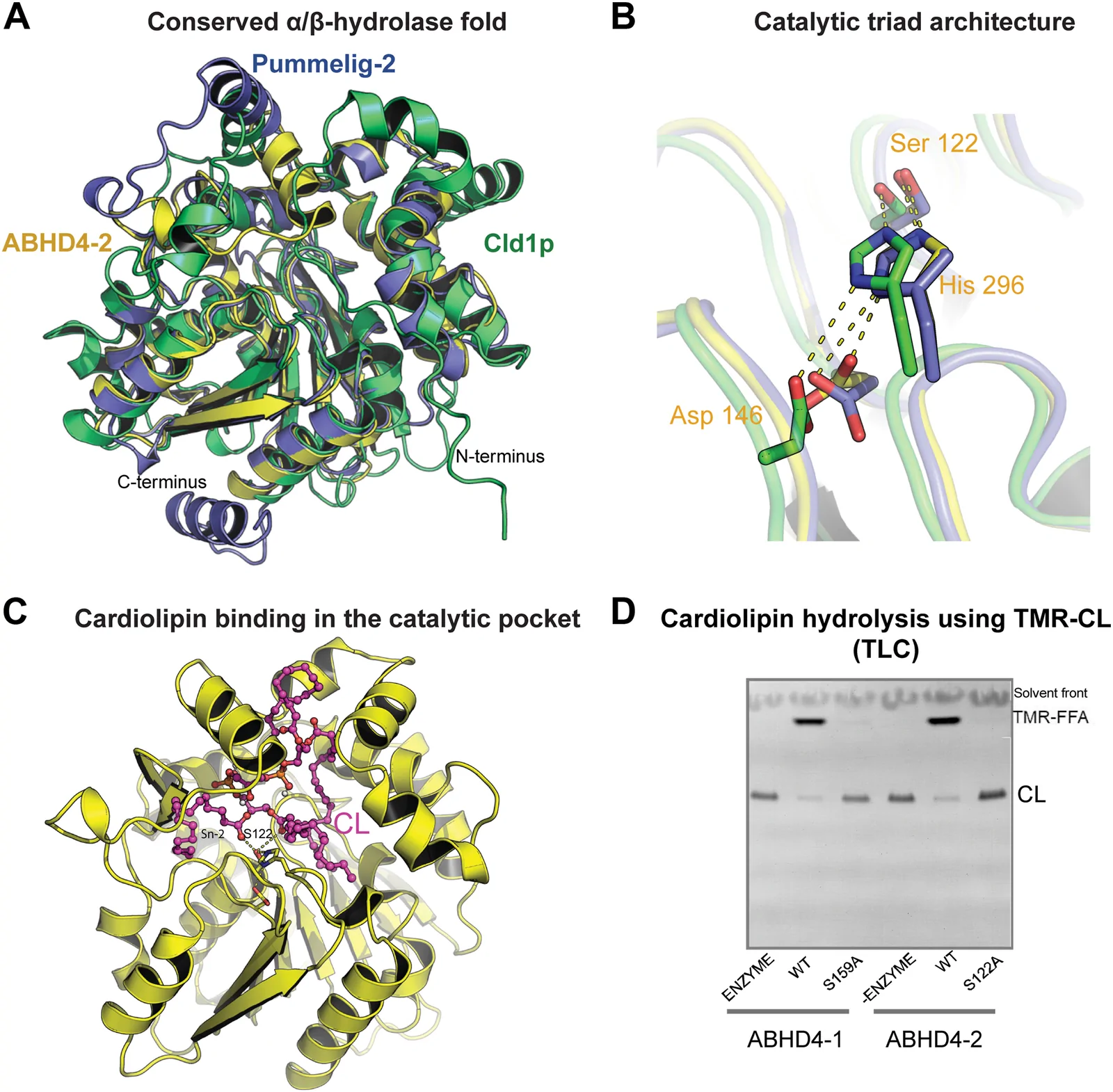
A conserved α/β-hydrolase fold and catalytic triad support CL lipase activity in ABHD4, Pummelig, and Cld1p. A: Structural alignment of AlphaFold-predicted models of ABHD4-2 (yellow), Pummelig-2 (blue), and yeast Cld1p (green) reveals a conserved α/β-hydrolase fold composed of a central β-sheet surrounded by α-helices. Structural differences are primarily observed in the N-terminal region and adjacent helical elements. B: Close-up view of the catalytic site showing conservation of the catalytic triad. In ABHD4-2, Ser122, Asp146, and His296 adopt a geometry consistent with nucleophilic catalysis. The catalytic serine (S122/S123/S230), aspartate (D146/D147/D392), and histidine (H296/H299/H424) residues are conserved across ABHD4, Pummelig, and Cld1p. C: Predicted cardiolipin binding within the catalytic pocket of ABHD4-2. CL (magenta) is accommodated within a hydrophobic cavity, with the glycerol backbone oriented toward the catalytic triad, consistent with positioning for acyl chain hydrolysis. D: CL binding pose validation by hydrolysis using TMR-labeled cardiolipin. The sn-2 position is labeled with TMR, whereas the remaining acyl chains are unlabeled. WT ABHD4-1 and ABHD4-2 generated TMR-labeled FFA, whereas catalytic mutants lost activity.

Alignment of AlphaFold structural models further revealed that ABHD4, Pummelig, and Cld1p share a conserved α/β-hydrolase fold, composed of eight central β-strands surrounded by α-helices (Fig. 7A). The main differences were observed in the N-terminal region and in the helical regions surrounding the catalytic core, which likely function as lid domains that regulate substrate access. Closer inspection of the active site regions identified a conserved catalytic triad of serine (S122/S123/S230), aspartate (D146/D147/D392), and histidine (H296/H299/H424) across the three proteins (Fig. 7B). In ABHD4-2, the catalytic residues Ser122, Asp146, and His296 are positioned for nucleophilic catalysis, as observed in other serine hydrolases (51, 52). Docking analysis indicates that CL binds within the catalytic pocket of ABHD4-2, with one *sn-2* acyl chain positioned near the catalytic Ser122 (Fig. 7C). The lipid is positioned within a hydrophobic pocket, with the glycerol backbone oriented toward the catalytic triad, thereby positioning the substrate for *sn-2* cleavage. Consistent with this binding mode, ABHD4-1 and ABHD4-2 released fluorescently tagged fatty acid from the *sn-2* position of TMR-CL (Fig. 7D). This structural modeling, together with biochemical findings, supports a conserved catalytic mechanism underlying CL lipase activity in ABHD4 and its homologs.

### ABHD4-2 catalytic activity reduces ΔΨm

To examine the effect of ABHD4-2 in cells, we generated doxycycline-inducible stable BA cell lines expressing WT ABHD4-2 or the catalytic mutant (S122A). Endogenous ABHD4 expression is undetectable in these BAs under basal conditions. In this system, both WT and mutant proteins localized to the mitochondria, consistent with their distribution in COS-7 cells, indicating that mitochondrial localization is independent of cell type (supplemental Fig. S14). This approach enabled controlled expression of ABHD4-2 to assess whether its catalytic activity influences mitochondrial function, as measured by quantifying ΔΨm using the JC-1 fluorescence assay in live BAs.

**Fig. 8:**
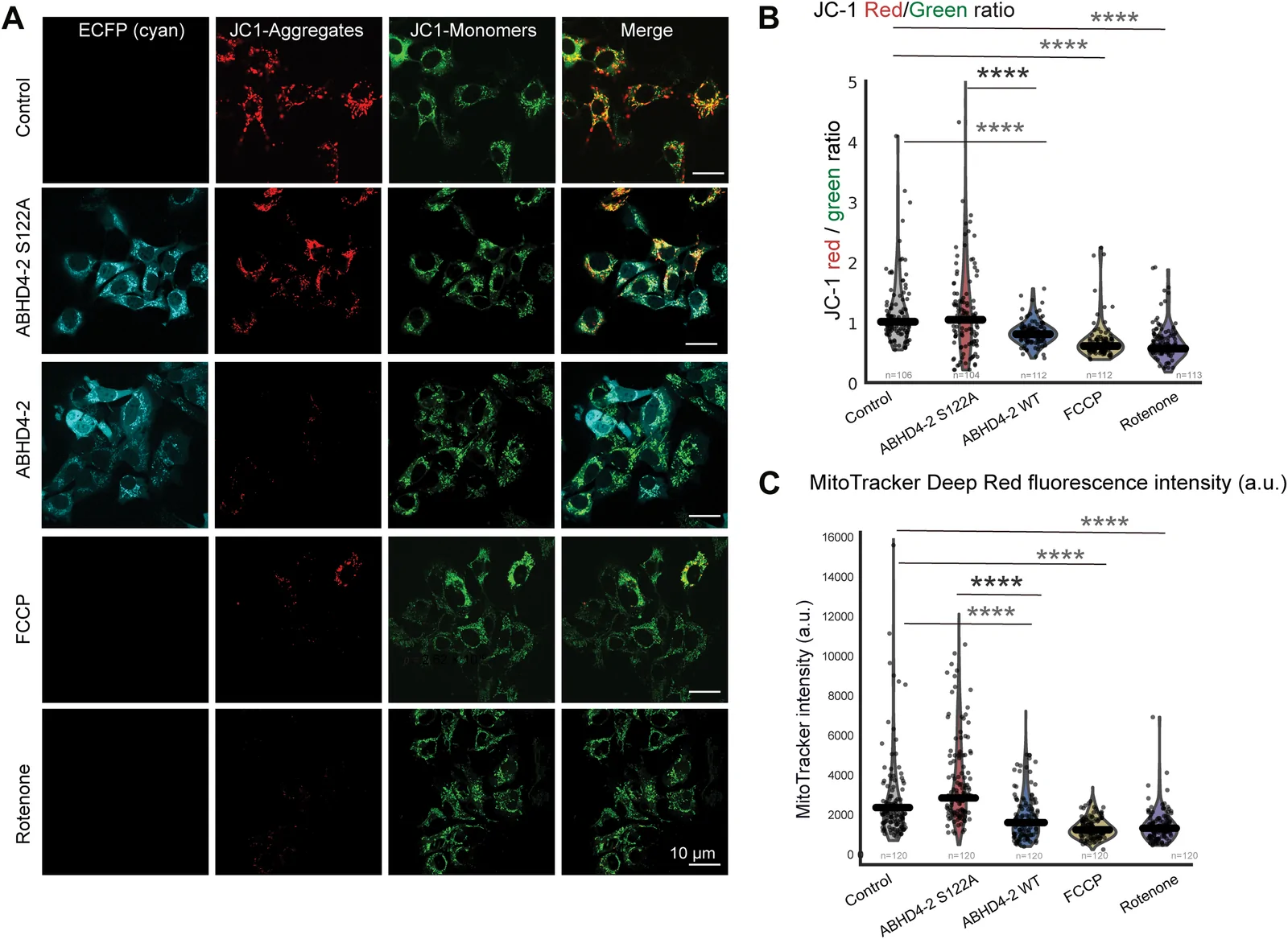
ABHD4-2 catalytic activity reduces mitochondrial membrane potential (ΔΨm). A: Representative confocal images of brown adipocytes expressing ABHD4 WT or the catalytic mutant S122A and stained with JC-1. JC-1 aggregates (red) correspond to polarized mitochondria, and JC-1 monomers (green) reflect depolarized mitochondria. ECFP (cyan) identifies ABHD4-expressing cells. FCCP and rotenone were included as depolarizing controls under comparable conditions. Images were acquired using identical settings across all conditions. Scale bar, 10 μm. B: Quantification of mitochondrial membrane potential based on the JC-1 red/green ratio calculated on a per-cell basis. ABHD4 WT shows a statistically significant reduction in the red/green ratio relative to control cells, and the S122A mutant retains a higher ratio. FCCP and rotenone produce the expected decrease. Each point represents a single cell (∼100 cells per condition from three independent experiments), with horizontal bars indicating median values. Statistical significance was assessed using two-sided Mann-Whitney U test (∗∗∗∗*P* < 0.0001). C: Quantification of MitoTracker Deep Red intensity on a per-cell basis. ABHD4 WT is associated with reduced signal intensity relative to control cells, and the S122A mutant shows higher intensity compared with WT. FCCP and rotenone similarly reduce MitoTracker signal. Each point represents a single cell (∼100 cells per condition from three independent experiments), with horizontal bars indicating median values. Statistical significance was assessed using two-sided Mann-Whitney U test (∗∗∗∗*P* < 0.0001).

Control cells exhibited robust JC-1 aggregate (red) fluorescence, indicating a polarized mitochondrial state. As expected, treatment with FCCP or rotenone reduced red fluorescence and increased the JC-1 monomer (green) signal, validating effective mitochondrial depolarization (Fig. 8A). Quantification of the JC-1 red/green ratio on a per-cell basis showed that expression of ABHD4-2 WT significantly reduced mitochondrial membrane potential (*P* < 0.0001) (Fig. 8B). In contrast, cells expressing the catalytic mutant ABHD4-2 S122A maintained a higher red/green ratio compared with WT (*P* < 0.0001), indicating that this effect depends on catalytic activity. Approximately 100 cells per condition were analyzed in each independent experiment. Three independent experiments were performed, resulting in ∼300 cells analyzed per condition, and statistical significance was evaluated using the Mann-Whitney U test. MitoTracker Deep Red staining, which is also sensitive to mitochondrial membrane potential, confirmed that ABHD4-2 overexpression reduces mitochondrial membrane potential in a manner requiring catalytic activity (Fig. 8C). Collectively, these results indicate that ABHD4-2 catalytic activity can reduce mitochondrial membrane potential.

## Discussion

CL is a central mitochondrial phospholipid that maintains membrane architecture and supports stress signaling across mitochondrial membranes. While CL metabolism has been extensively studied at the IMM, the enzymatic basis of CL turnover at the OMM has remained incompletely defined. Here, we identify ABHD4 and its homolog Pummelig as conserved cardiolipin hydrolases and show that their shorter isoforms localize to the cytosolic face of the OMM, defining a spatially distinct site of CL metabolism.

ABHD4 and Pummelig are expressed as splice variants that differ at the N terminus and exhibit distinct subcellular targeting. The longer isoforms, ABHD4-1 and Pummelig-1, contain a putative LD-targeting region and localize to LDs, whereas the shorter isoforms, ABHD4-2 and Pummelig-2, lack this region and instead localize to mitochondria. This isoform-specific distribution is consistent with the enrichment of ABHD4-2 in oxidative tissues and suggests that alternative splicing directs ABHD4 to distinct lipid environments. Such spatial segregation likely enables compartment-specific regulation of lipid metabolism and provides a framework for linking lipid remodeling to organelle-specific function.

Biochemical analyses demonstrate that ABHD4 catalyzes the stepwise deacylation of CL to generate MLCL and DLCL. This mechanism resembles ABHD18 (17), the recently identified mammalian CL phospholipase and functional homolog of yeast Cld1p (13), which deacylates CL at the matrix side of the IMM. ABHD4-2 generates the same products, MLCL and DLCL, but acts from the outer membrane instead. Proteinase K protection places ABHD4-2 on the cytosolic face of the OMM, positioning it to access OMM-associated CL pools exposed during apoptosis and mitophagy (8, 21, 22, 23, 24, 25). ABHD4-2 and ABHD18 hydrolyze CL at distinct membranes, the OMM and IMM, respectively, highlighting a compartment-specific role for CL hydrolysis within mitochondria. In addition to direct CL hydrolysis, the presence of CL in phospholipid bilayers promotes ABHD4 activity toward other phospholipids, generating LPC and LPE species, suggesting that membrane composition contributes to substrate recognition, with CL functioning as both the preferred substrate and a regulator of enzymatic activity. Together, these findings establish ABHD4 as a CL-directed phospholipase that produces MLCL and DLCL as key intermediates in CL remodeling.

Our extended phospholipid vesicle assays suggest that membrane composition is an important determinant of ABHD4-2 activity. Although ABHD4-2 hydrolyzes CL most efficiently, it hydrolyzes PG and PC only in CL-containing bilayers, indicating that CL contributes to substrate recognition beyond serving simply as a phospholipid substrate. Biophysically, CL alters membrane packing and preferentially partitions into curved membrane regions (61, 62). These membrane properties can influence peripheral protein-membrane interactions (63) and may facilitate ABHD4-2 association with the membrane, thereby enhancing access to neighboring phospholipid substrates at the membrane interface, consistent with established principles of phospholipase interfacial catalysis (64, 65, 66).

The identification of ABHD4-2 as an OMM-targeted, CL-selective hydrolase has implications for understanding CL functions at the OMM. First, ABHD4-mediated CL hydrolysis at the OMM provides a plausible mechanism for modulating mitochondrial stress responses, particularly apoptosis (9, 67, 68), anoikis (28), and mitophagy (8). ABHD4 mediates anoikis in certain contexts (28, 30), although the molecular mechanism underlying this activity remains unclear. ABHD4-dependent anoikis requires catalytic activity but is independent of the NAPE hydrolysis pathway (30). Our results show that mitochondrial ABHD4-2 activity reduces ΔΨm, an event that often precedes programmed cell death (69, 70). Second, CL regulates several processes that could contribute to this loss of membrane potential, including membrane curvature, lipid packing, and protein-lipid interactions (1, 3, 4). While one might expect that depletion of OMM CL would reduce susceptibility to programmed cell death, the products of CL hydrolysis, MLCL and DLCL, are known to function as signaling intermediates in programmed cell death pathways (9, 11, 71). Third, the existence of distinct ABHD4 isoforms supports a model in which lipid metabolism is coordinated across organelles via alternative splicing and compartment-specific targeting. This compartmentalization may enable localized regulation of CL-dependent signaling at the mitochondrial surface.

Key questions remain unresolved and are under current investigation. The physiological role of ABHD4-1 at LDs remains unknown. We previously reported that although ABHD4-1 localizes to LDs, it does not activate ATGL-dependent lipolysis (72), distinguishing it from the established ATGL co-activator CGI-58/ABHD5. For ABHD4-2, whether the enzyme exhibits selectivity toward defined CL molecular species or oxidized forms is unknown (73). The impact of ABHD4-dependent CL hydrolysis on the recruitment or activation of apoptotic and mitophagic machinery also remains to be established. Whether physiological stimuli, including fasting, nutrient deprivation, or high-fat diet, regulate ABHD4 isoform expression, alternative splicing, or subcellular localization also remains to be determined. In addition, the mechanisms governing ABHD4 targeting and regulation under basal versus stress conditions require further investigation.

In summary, ABHD4 and its *Drosophila* homolog Pummelig constitute a conserved family of mitochondrial CL hydrolases. Alternative splicing generates isoforms that are differentially targeted to LDs or mitochondria. The mitochondrial isoform, ABHD4-2, resides on the cytosolic surface of the OMM and is structurally and mechanistically related to Cld1p and ABHD18. ABHD4-2 catalyzes the sequential deacylation of CL to MLCL and DLCL through a conserved active-site serine. Although ABHD4-2 also hydrolyzes other phospholipids, hydrolysis of these substrates depends on the presence of CL within the membrane bilayer, whereas CL remains the preferred substrate. ABHD4-2 catalytic activity is associated with reduced mitochondrial membrane potential, linking CL hydrolysis at the OMM to mitochondrial bioenergetic status. Together, these findings establish ABHD4-2 as an OMM-localized, CL-preferential phospholipase and identify the OMM as a site of CL hydrolysis.

## Data availability

The data supporting the findings of this study are available in the article and the supplementary information files. Additional data and custom Python analysis code are available from the corresponding author upon request.

## Supplemental data

This article contains supplementary data, including immunoblot validation of ABHD4 and Pummelig isoforms, structural comparison of ABHD4 isoforms with related homologs, isoform-specific localization of Pummelig, and additional analyses of Pummelig activity and TLC-based lipid hydrolysis.

## Conflict of interest

The authors declare that they have no conflicts of interest with the contents of this article.
