# Supplementary material for: ABHD4-2: a cardiolipin-selective phospholipase of the outer mitochondrial membrane
Source: J Lipid Res. 2026 Aug 6;67(9):101119. doi: 10.1016/j.jlr.2026.101119 (PMC13572076; doi:10.1016/j.jlr.2026.101119)
Supplement: Supplementary file 1 [file mmc1.pdf]

Supplemental Data

ABHD4-2: a cardiolipin-selective phospholipase of the outer mitochondrial membrane

Raja Narayanasamy[1,‡], Matthew A. Sanders[1,‡], Huamei Zhang[1], Marta Moreno-Torres[2,†], Vanesa D. Ramseyer[1], Hong-Guang Wei[1], Nils J. Færgeman[2], Christopher V. Kelly[3,5], and James G. Granneman[1,4,5,*]

[1] Center for Molecular Medicine and Genetics, Wayne State University School of Medicine, Detroit, MI, USA

[2] Department of Biochemistry and Molecular Biology, University of Southern Denmark, Odense M, Denmark

[3] Department of Physics and Astronomy, Wayne State University, Detroit, MI, USA

[4] Center for Integrative Metabolic and Endocrine Research, Wayne State University School of Medicine, Detroit, MI, USA

[5] Barber Center for Multiscale Systems Biology, Wayne State University, Detroit, MI, USA

[*]For correspondence: James G. Granneman, Center for Molecular Medicine and Genetics, Wayne State University School of Medicine, Detroit, MI 48201, USA. Tel: +1 313-577-5629. Email: jgranne@med.wayne.edu

[‡]These authors contributed equally to this work.

[†]Current address for Marta Moreno-Torres: Department of Biochemistry and Molecular Biology, University of Valencia, Valencia, Spain; Experimental Hepatology Joint Research Unit, Health Research Institute La Fe (IIS La Fe), Valencia, Spain; Centro de Investigación Biomédica en Red de Enfermedades Hepáticas y Digestivas (CIBERehd), Instituto de Salud Carlos III, Madrid, Spain.

Short title: ABHD4-2 is an outer mitochondrial membrane phospholipase

Funding: This work was supported by the National Institutes of Health Grants DK076229 and DK062292 and by the Barber Center for Multiscale Systems Biology. The project described was supported by Grant Number P30DK020572 (MDRC) from the National Institute of Diabetes and Digestive and Kidney Diseases. M. M. T. acknowledges the postdoctoral fellowship funded by the Danish Diabetes Academy and the grant RYC2021-031346-I funded by MICIU/AEI/10.13039/501100011033 and by the European Union Next Generation EU/PRTR.

Abbreviations: ABHD4, alpha/beta hydrolase domain-containing protein 4; Cld1p, cardiolipin deacylase 1 protein; ABHD5, alpha/beta hydrolase domain-containing protein 5; ABHD18, alpha/beta hydrolase domain-containing protein 18; PLIN5, perilipin 5; PNPLA8, patatin-like phospholipase domain-containing protein 8; iPLA^{2}γ, Ca^{2+}-independent phospholipase A^{2}γ; NDUFS1, NADH:ubiquinone oxidoreductase core subunit S1; MECR, mitochondrial trans-2-enoyl-CoA reductase; CL, cardiolipin; LCL, lysocardiolipin; MLCL, monolysocardiolipin; DLCL, dilysocardiolipin; OMM, outer mitochondrial membrane; IMM, inner mitochondrial membrane; IMS, intermembrane space; LD, lipid droplet; BAT, brown adipose tissue; NAPE, N-acylphosphatidylethanolamine; LPC, lysophosphatidylcholine; LPE, lysophosphatidylethanolamine; FFA, free fatty acid; LC–MS, liquid chromatography–mass spectrometry; TLC, thin-layer chromatography; $\Delta\Psi m$, mitochondrial membrane potential.

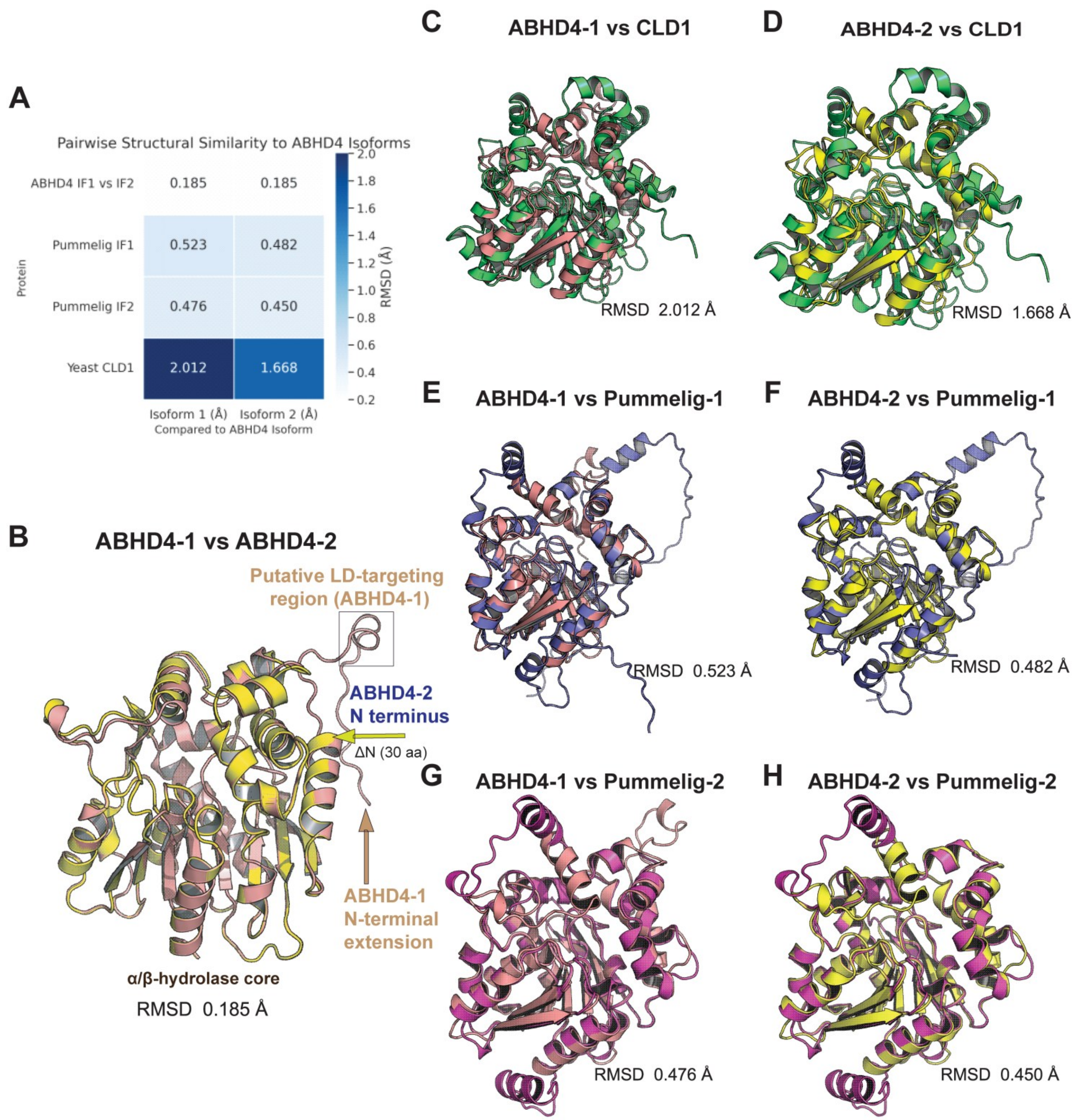

Supplemental Fig. S1: Structural comparison of predicted models of ABHD4 isoforms with Cld1p and Pummelig homologs.

(A) Pairwise RMSD heatmap comparing ABHD4 isoforms with homologs from *Drosophila melanogaster* (Pummelig) and *Saccharomyces cerevisiae* (Cld1p). ABHD4-1 and ABHD4-2 show high structural similarity with minimal deviation (0.185 Å). Cld1p shows comparable structural similarity to both ABHD4 isoforms (RMSD 1.668–2.012 Å), with

slightly closer alignment to ABHD4-2 (1.668 Å) than to ABHD4-1 (2.012 Å), consistent with the N-terminal truncation in ABHD4-2.

(B) Superimposition of ABHD4-1 (salmon) and ABHD4-2 (yellow). The two isoforms adopt nearly identical structures (RMSD 0.185 Å). The isoforms differ only at the N terminus; ABHD4-2 lacks the ~30-residue N-terminal extension present in ABHD4-1, whereas the α/β-hydrolase core is conserved.

(C–D) Structural alignment of ABHD4 isoforms with yeast Cld1p (green). Cld1p shows clear structural similarity to both ABHD4 isoforms (RMSD 2.012 Å and 1.668 Å), with conservation of the central α/β-hydrolase fold.

(E–F) Structural alignment with Pummelig-1 (blue). Low RMSD values (0.523 Å and 0.482 Å) indicate strong structural conservation, supporting a close relationship between ABHD4 and Pummelig proteins.

(G–H) Structural alignment with Pummelig-2 (magenta). Similar RMSD values (0.476 Å and 0.450 Å) further support this conservation. All RMSD values represent Cα atom alignments.

Across all comparisons, the α/β-hydrolase core is conserved, whereas variability is largely confined to the N-terminal region and surface-exposed loops. The structural similarity between ABHD4, Pummelig, and Cld1p suggests a conserved α/β-hydrolase framework across species.

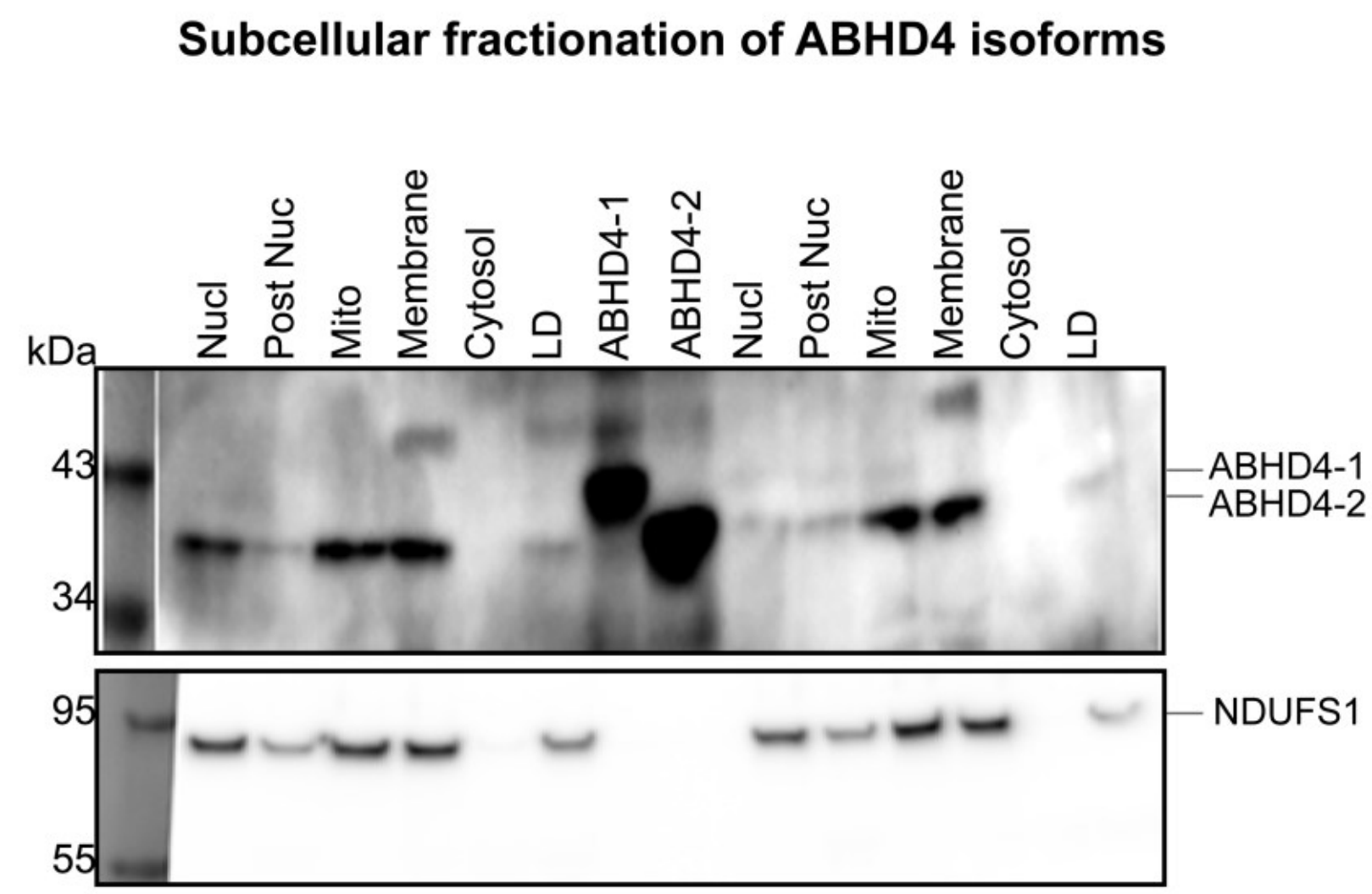

Supplemental Fig. S2: ABHD4-2 is enriched in mitochondrial and membrane fractions.

Brown adipose tissue subcellular fractions were separated and analyzed by immunoblotting using an anti-ABHD4 antibody. Fractions included nuclear (Nuc), post-nuclear supernatant (Post-nuclear), mitochondrial (Mito), membrane, cytosolic, and lipid droplet (LD) fractions. Lysates from COS-7 cells expressing ABHD4-1 and ABHD4-2 were used as controls. NDUFS1 is shown as a mitochondrial marker.

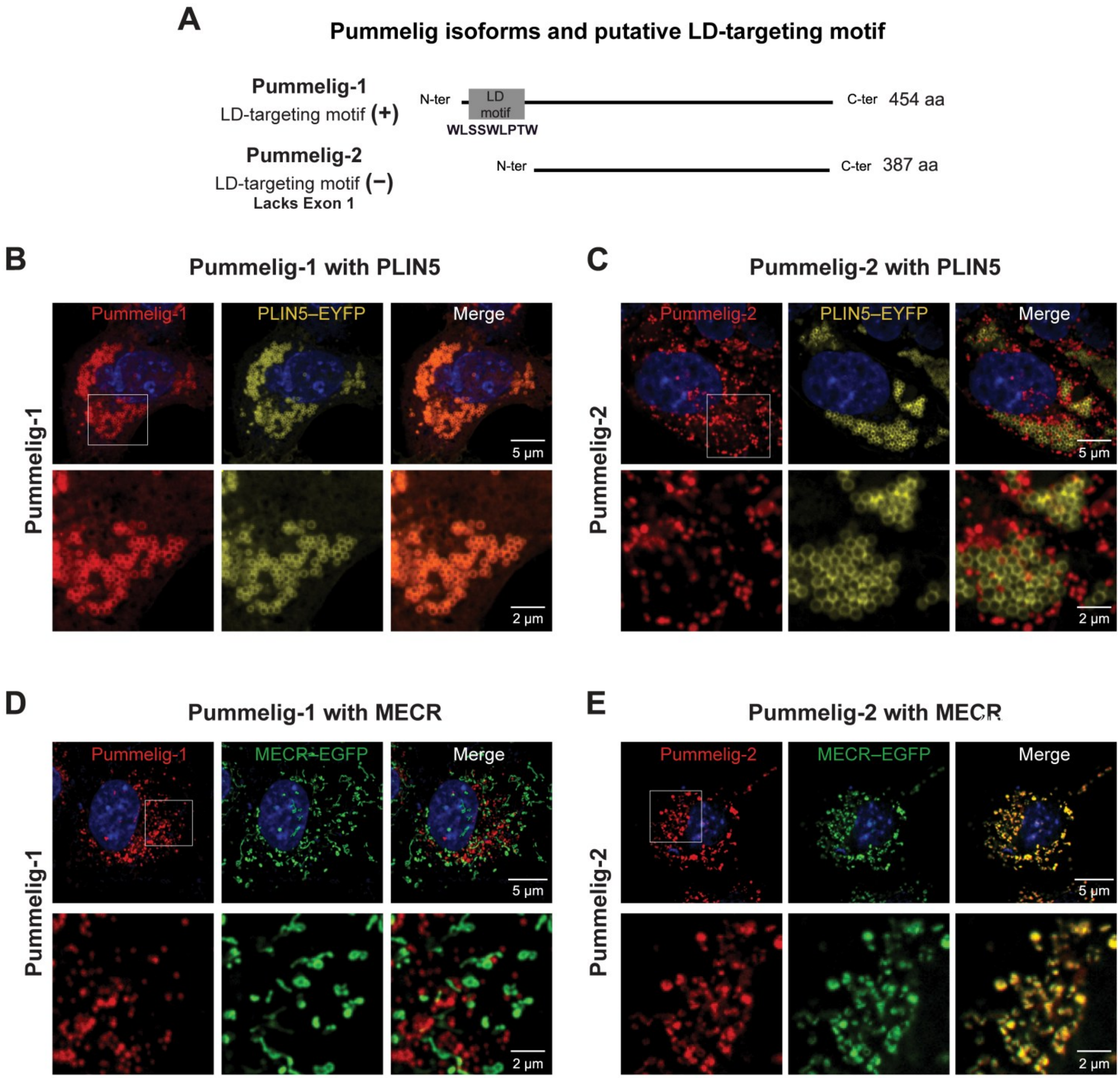

Supplemental Fig. S3: Isoform-specific subcellular localization of Pummelig in COS-7 cells.

Confocal imaging of COS-7 cells transiently co-transfected with fluorescently tagged isoforms and organelle-specific markers. Cells were incubated overnight with 200 µM oleic acid (16–20 h) prior to imaging. Pummelig-1 (long isoform) and Pummelig-2 (short isoform) show distinct subcellular distributions, consistent with the pattern observed for ABHD4 (Fig. 3).

(A) Domain organization of isoforms. The long isoform, Pummelig-1 (454 aa), contains an N-terminal region with a putative lipid droplet (LD)-targeting motif, whereas the short isoform, Pummelig-2 (387 aa), lacks this region.

(B–C) Isoforms and lipid droplets. COS-7 cells expressing mCherry-tagged constructs (red) were coexpressed with PLIN5-EYFP (Perilipin 5; LD marker). Pummelig-1 is observed in association with PLIN5-positive LD structures, whereas Pummelig-2 shows minimal overlap.

(D–E) Isoforms and mitochondria. COS-7 cells expressing mCherry-tagged constructs (red) were coexpressed with MECR-EGFP (mitochondrial trans-2-enoyl-CoA reductase; mitochondrial marker). Pummelig-2 is observed in mitochondrial regions, whereas Pummelig-1 shows comparatively limited overlap, consistent with isoform-dependent mitochondrial association. Lower panels show magnified views of the corresponding regions above. Scale bars, 5 µm (top panels) and 2 µm (zoom panels).

Isoform-specific localization of ABHD4 and Pummelig using LipidTOX, PLIN5, and MECR

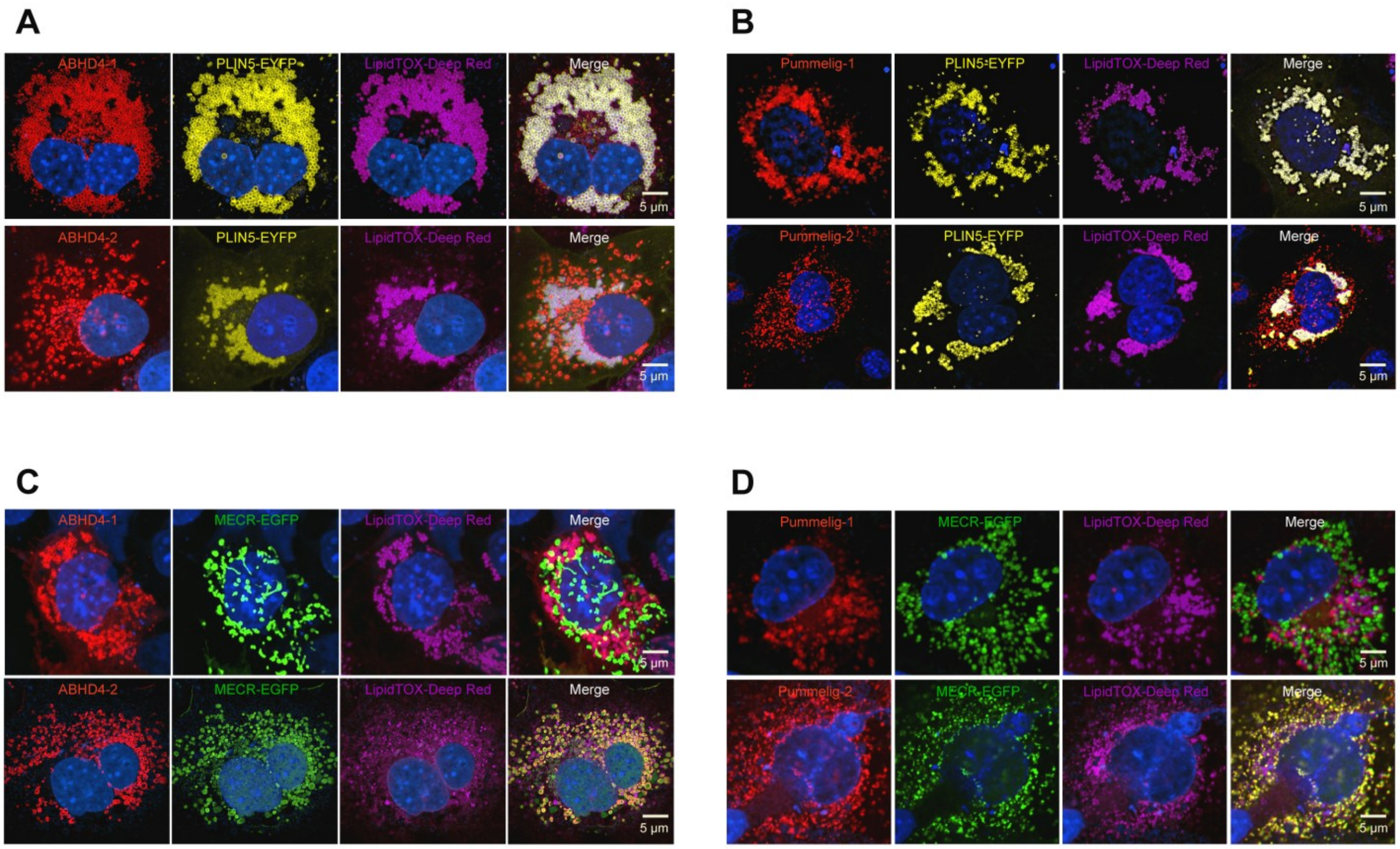

Supplemental Fig. S4: Isoform-specific localization of ABHD4 and Pummelig using PLIN5, MECR, and LipidTOX.

Confocal imaging of COS-7 cells expressing ABHD4 and Pummelig isoforms with lipid droplet and mitochondrial markers, providing additional validation of Fig. 3 and Fig. S3.

(A–B) ABHD4-1 and Pummelig-1 associate with lipid droplets, as indicated by colocalization with PLIN5-EYFP and LipidTOX. ABHD4-2 and Pummelig-2 show minimal overlap with lipid droplet markers.

(C–D) ABHD4-2 and Pummelig-2 localize to mitochondria, as indicated by colocalization with MECR-EGFP, whereas ABHD4-1 and Pummelig-1 show limited mitochondrial association. Scale bars, 5 µm.

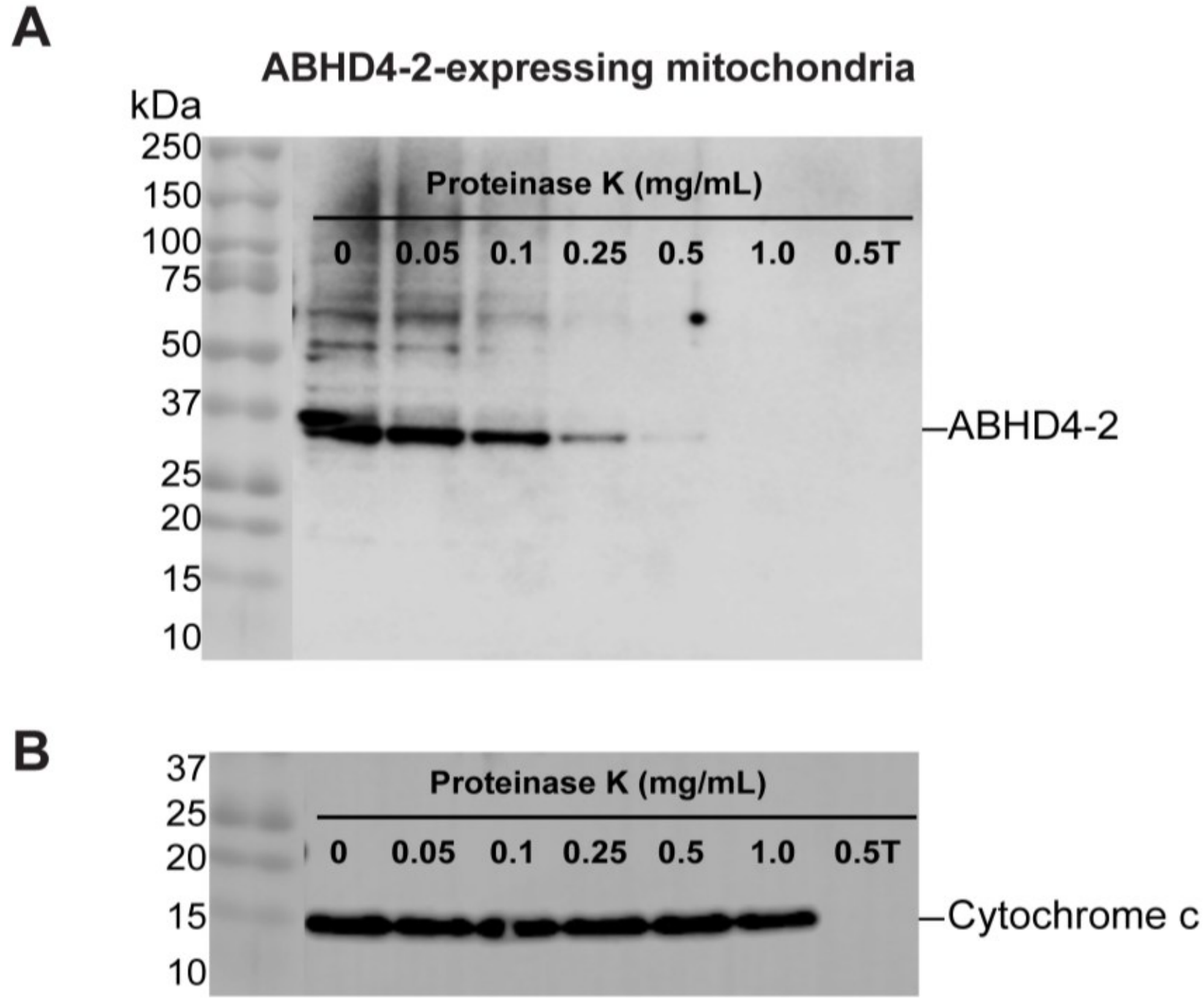

Supplemental Fig. S5: Proteinase K accessibility of ABHD4-2 relative to the IMS marker cytochrome c.

Mitochondria isolated from COS-7 cells expressing untagged ABHD4-2 were incubated with increasing concentrations of proteinase K (0–1.0 mg/mL), with or without 0.5% Triton X-100 (0.5T). Immunoblots were probed for (A) ABHD4-2 and (B) cytochrome c, an intermembrane space marker.

(A) ABHD4-2 was progressively degraded with increasing proteinase K concentration and was undetectable in the presence of Triton X-100.

(B) Cytochrome c remained protected across all proteinase K concentrations in intact mitochondria and was degraded only in the presence of Triton X-100. These results identify ABHD4-2 on the cytosolic surface of the outer mitochondrial membrane, distinct from the IMS-protected cytochrome c.

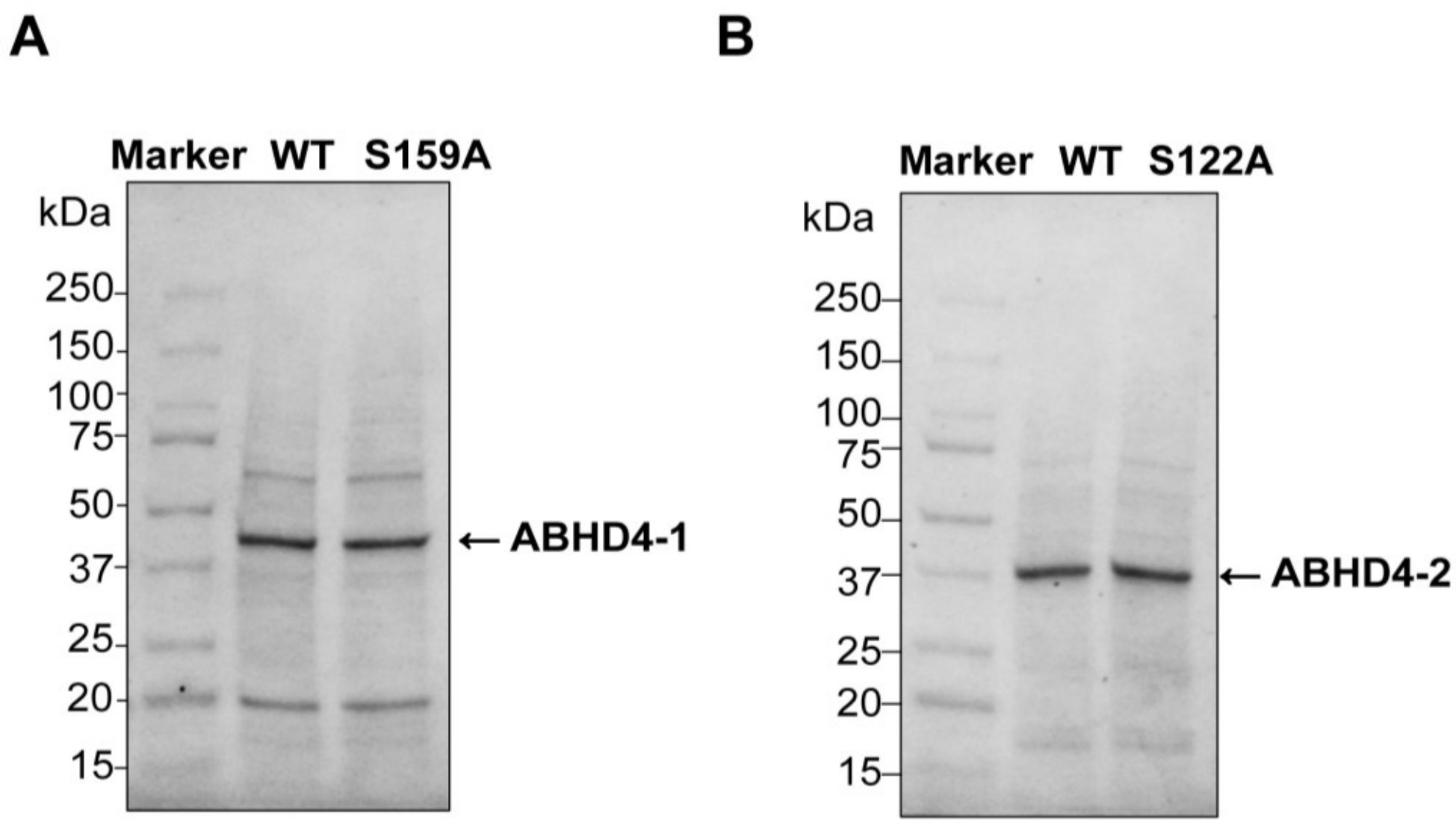

Supplemental Fig. S6: Purity of recombinant ABHD4 proteins.

Recombinant ABHD4-1 (WT and S159A) and ABHD4-2 (WT and S122A) were expressed as octa-histidine-tagged proteins in High Five insect cells via baculovirus infection and purified by Co^{2+} affinity resin chromatography. Coomassie Brilliant Blue-stained SDS-PAGE of the purified (A) ABHD4-1 and (B) ABHD4-2 proteins show a band at the expected molecular masses for each protein, confirming the purity of the preparations used for subsequent biochemical assays.

A

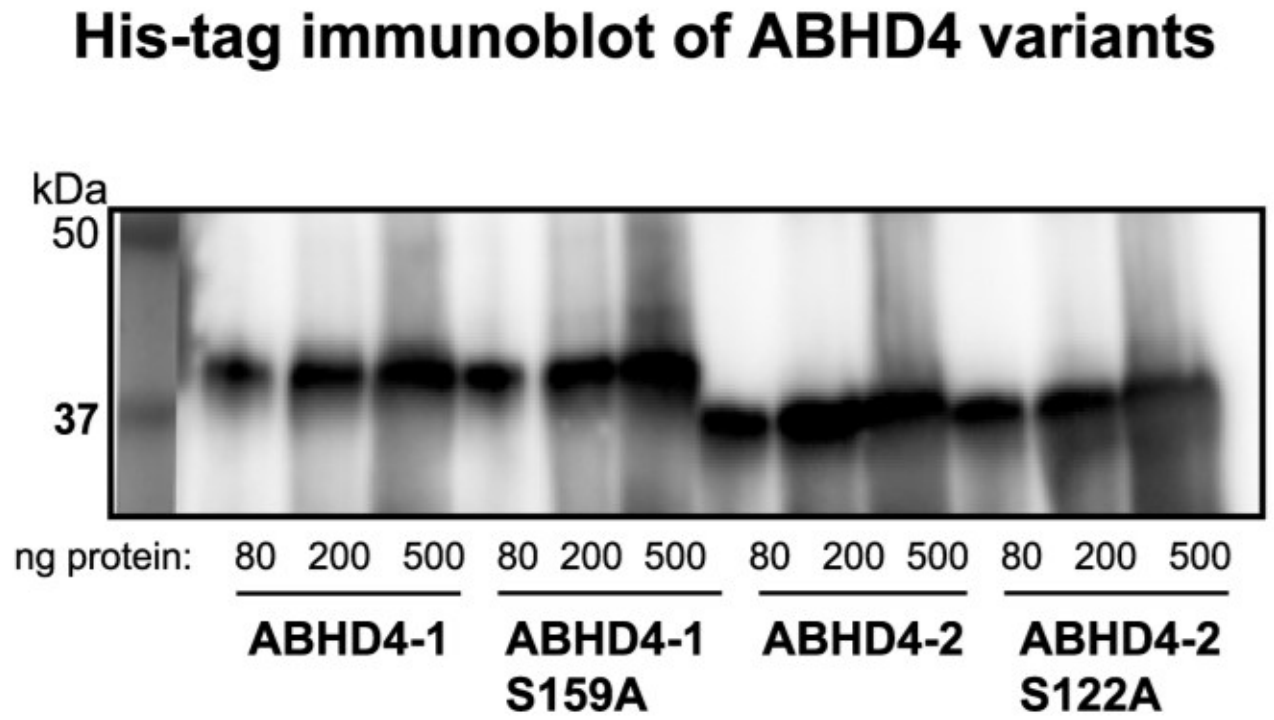

B

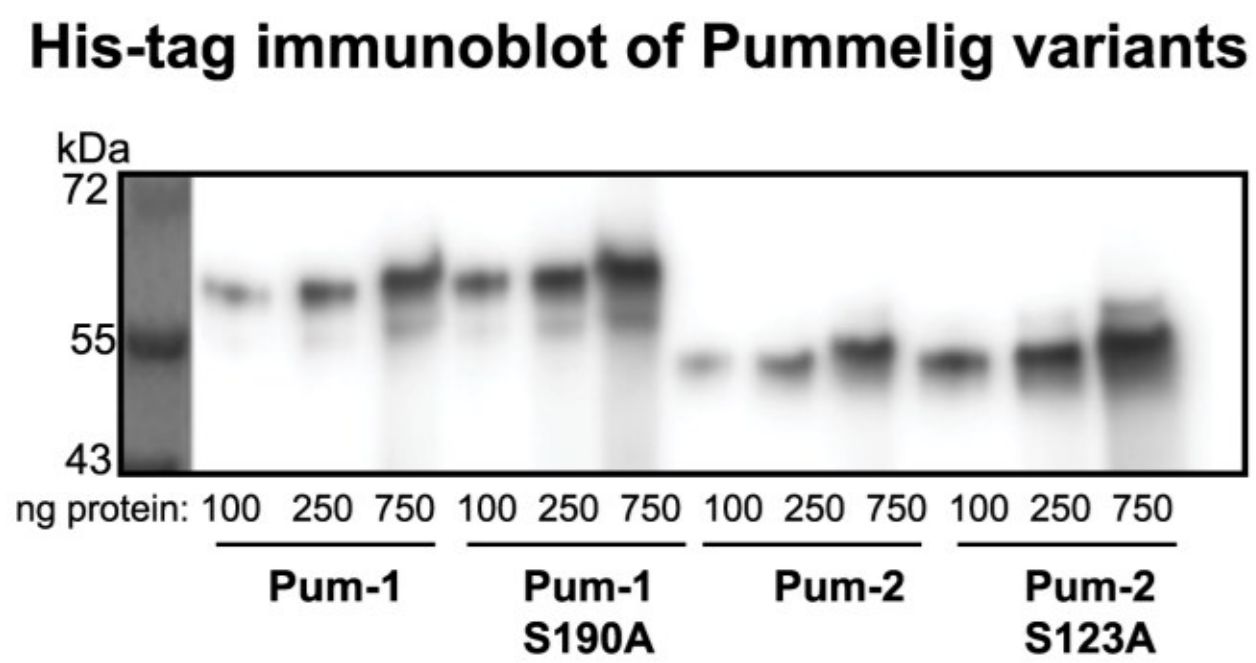

Supplemental Fig. S7: Immunoblot validation of recombinant ABHD4 and Pummelig isoforms and catalytic mutants.

(A) His-tag immunoblot of recombinant ABHD4 isoforms and catalytic mutants (S159A and S122A) loaded at increasing amounts (80, 200, and 500 ng).

(B) His-tag immunoblot of Pummelig isoforms (Pum-1 and Pum-2) and catalytic mutants (S190A and S123A) loaded at increasing amounts (100, 250, and 750 ng).

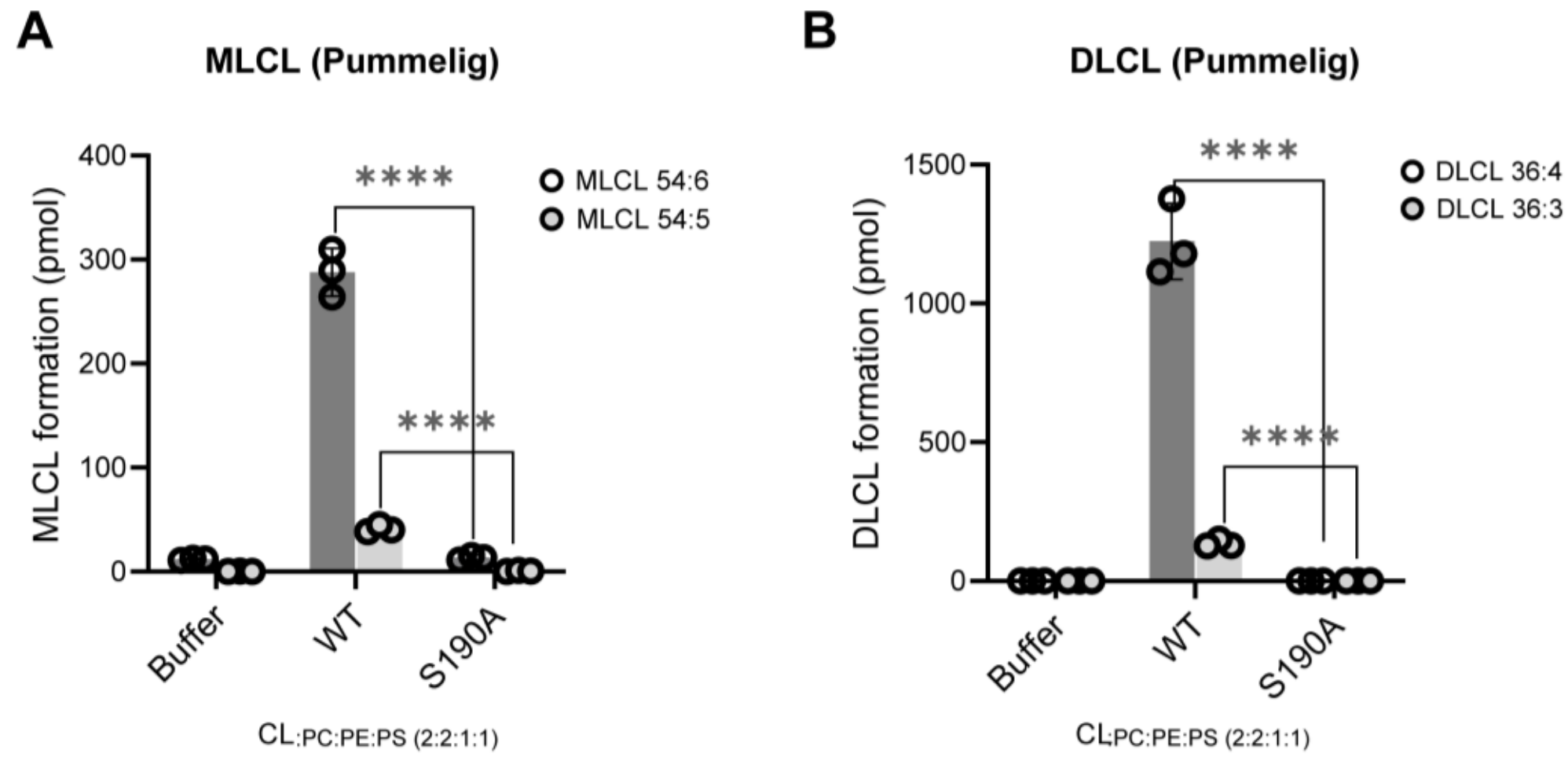

Supplemental Fig. S8: Pummelig catalyzes cardiolipin hydrolysis in a catalytic serine–dependent manner.

(A) MLCL formation by recombinant Pummelig-1 in CL-containing vesicles CL:PC:PE:PS (2:2:1:1). Wild-type proteins show MLCL formation, whereas the S190A mutant shows minimal activity.

(B) DLCL formation under the same conditions. Wild-type proteins produce DLCL, whereas the S190A mutant shows minimal activity. Statistical significance is indicated as ****$P < 0.0001$.

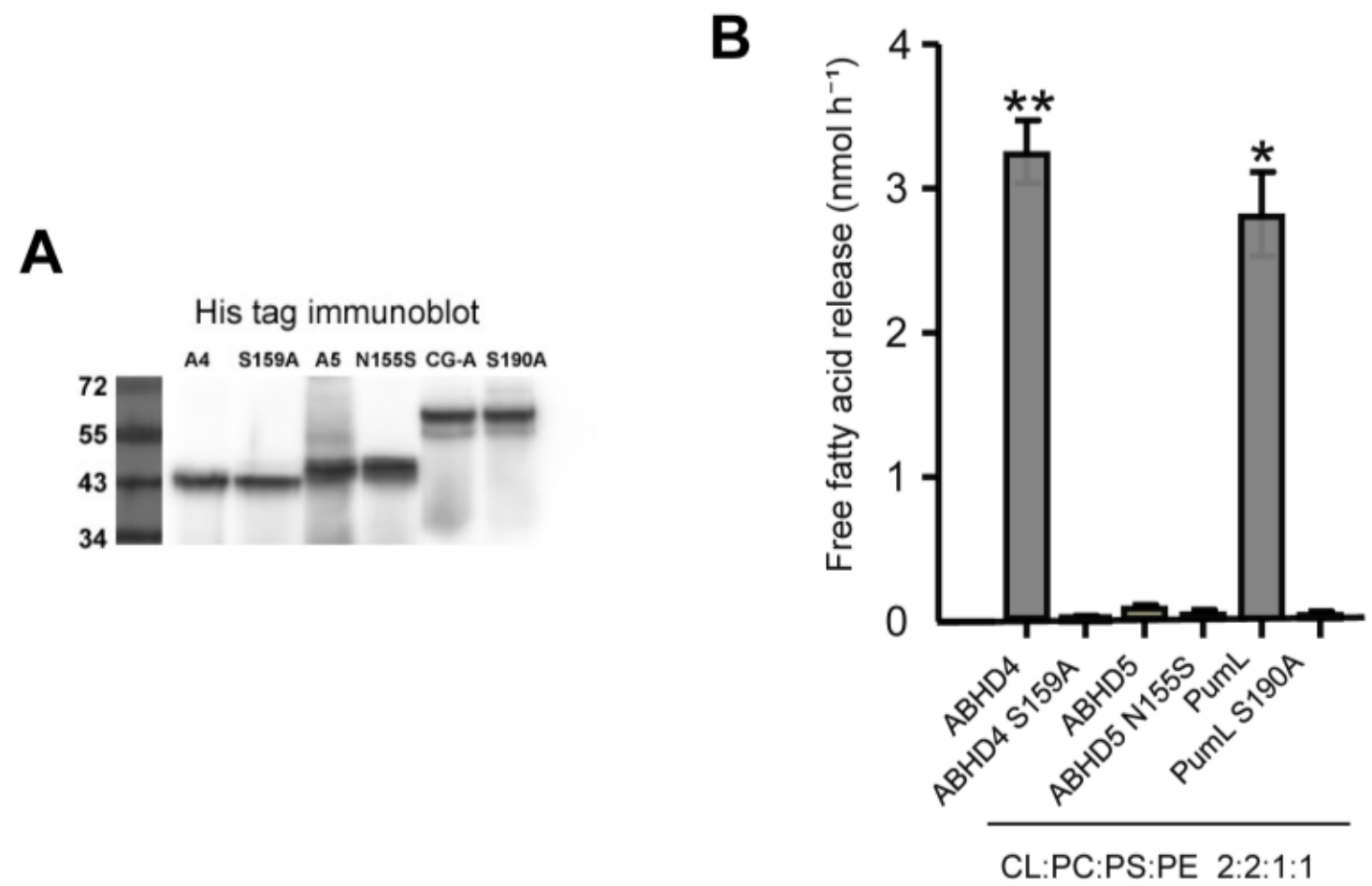

Supplemental Fig. S9: ABHD5 N155S mutation is not sufficient to restore cardiolipin lipase activity.

(A) His-tag immunoblot comparing ABHD4, ABHD5, and Pummelig proteins, including catalytic variants, to assess relative expression levels.

(B) Free fatty acid release was measured following incubation of recombinant proteins with cardiolipin-containing vesicles CL:PC:PE:PS (2:2:1:1). ABHD4 shows FFA release, whereas the catalytic mutant S159A shows minimal activity. ABHD5 shows no detectable activity under these conditions, and the N155S mutant shows no increase in FFA release. Pummelig (PumL) shows activity comparable to ABHD4. Data are presented as mean ± SD with individual data points representing independent reactions. Statistical significance is indicated as *$P < 0.05$ and **$P < 0.01$.

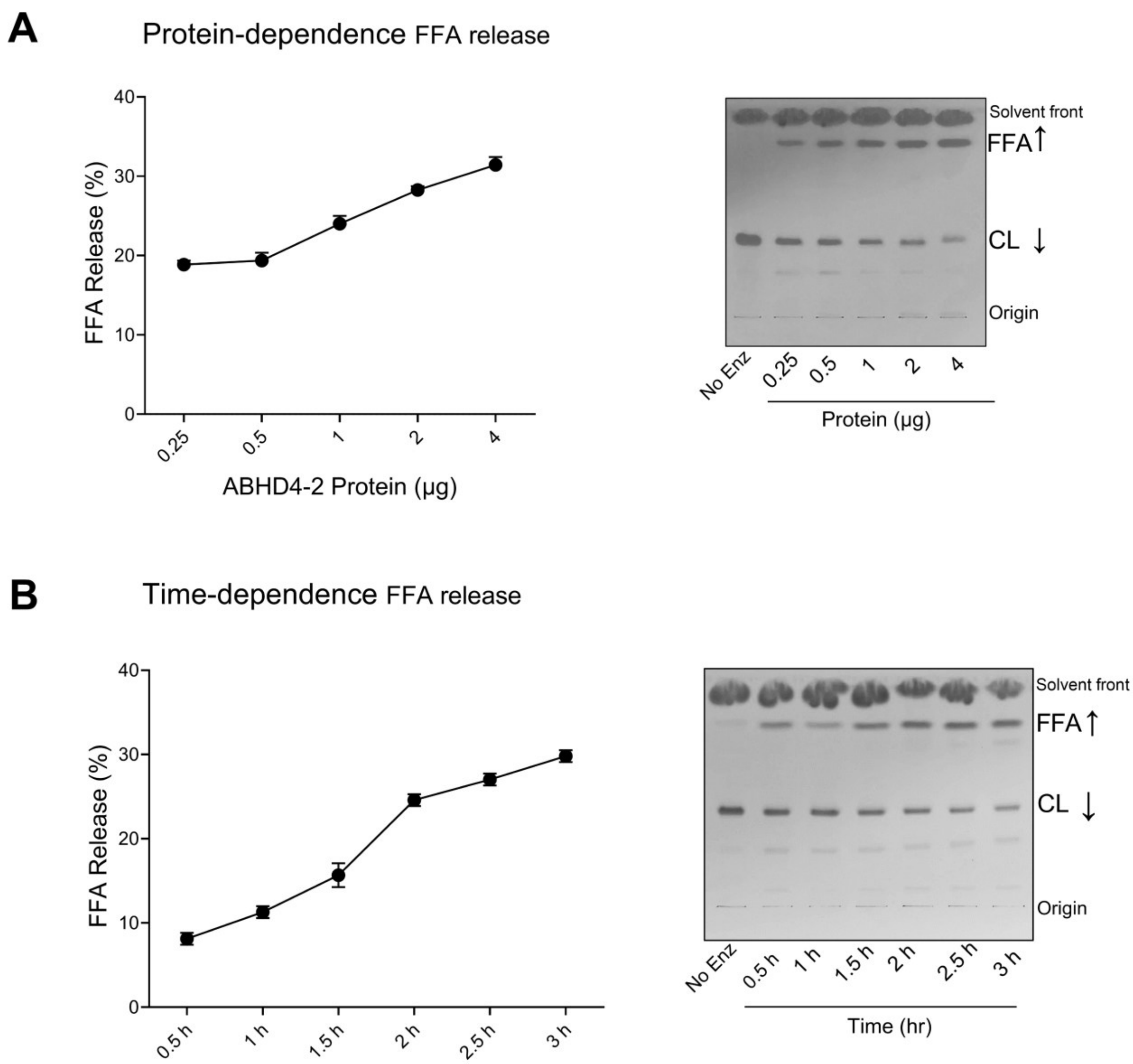

Supplemental Fig. S10: ABHD4-mediated CL hydrolysis is protein- and time-dependent.

(A) Protein-dependence lipase activity of ABHD4-2. Increasing amounts of purified ABHD4-2 (0.25–4 µg) were incubated with 50 µM native bovine heart cardiolipin substrates for 1 h, and free fatty acid release was quantified. FFA formation increased with enzyme concentration. Representative TLC analysis shows a corresponding increase in FFA and a decrease in CL.

(B) Time-dependence lipase activity of ABHD4-2. Purified ABHD4-2 (1 µg) was used to measure lipase activity over the indicated time points (0.5–3 h), showing a steady accumulation of FFA. TLC analysis showed that CL decreased over time, with a corresponding increase in FFA. Data are presented as mean ± SEM from at least three independent experiments.

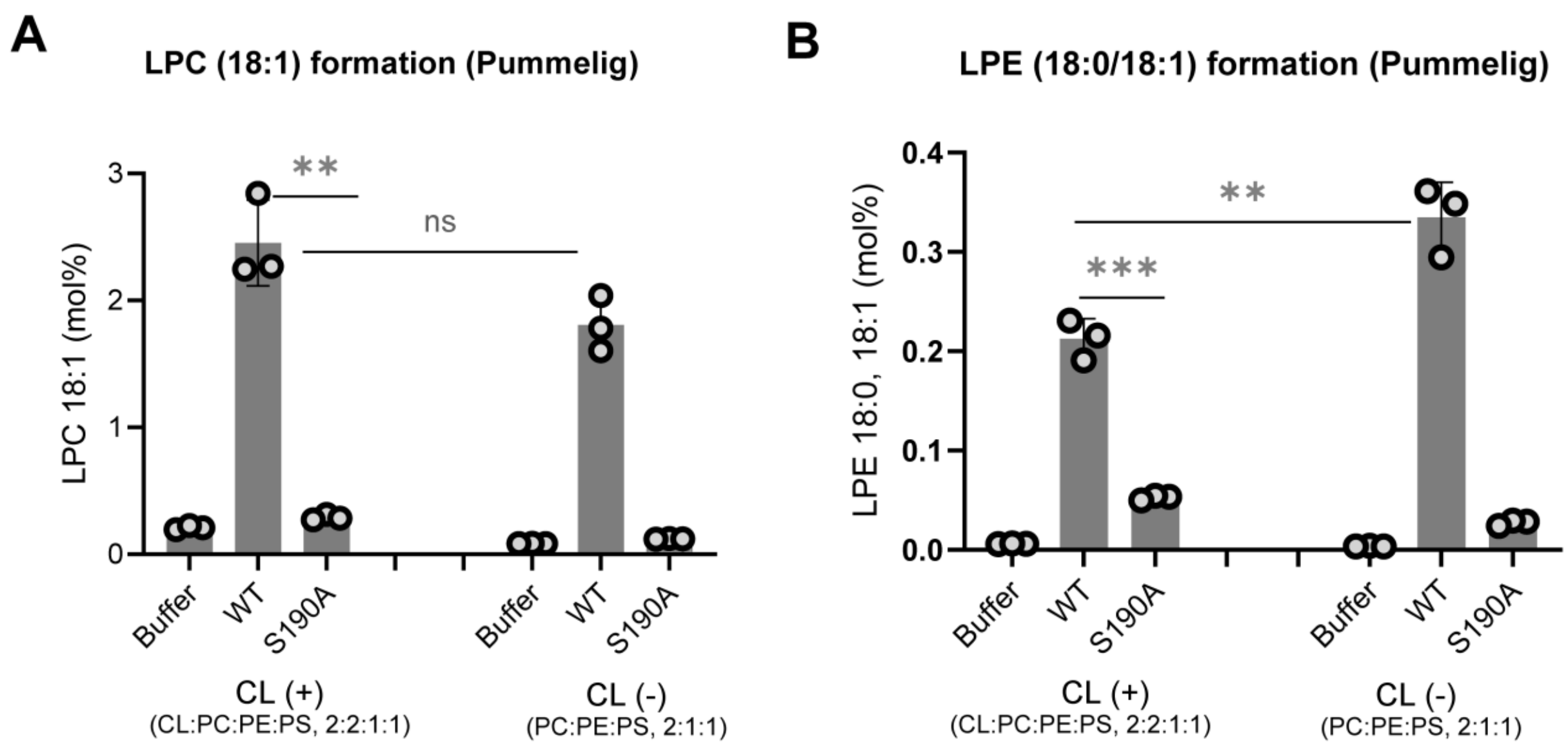

Supplemental Fig. S11: Pummelig promotes lysophospholipid formation.

(A) LPC (18:1) formation is observed with Pummelig-1 under both +CL and −CL conditions.

(B) LPE (18:0/18:1) formation is observed with Pummelig-1 under both +CL and −CL conditions. Data are presented as mean values with individual points representing independent reactions. Statistical significance is indicated as $^{**}P < 0.01$, $^{***}P < 0.001$; ns, not significant.

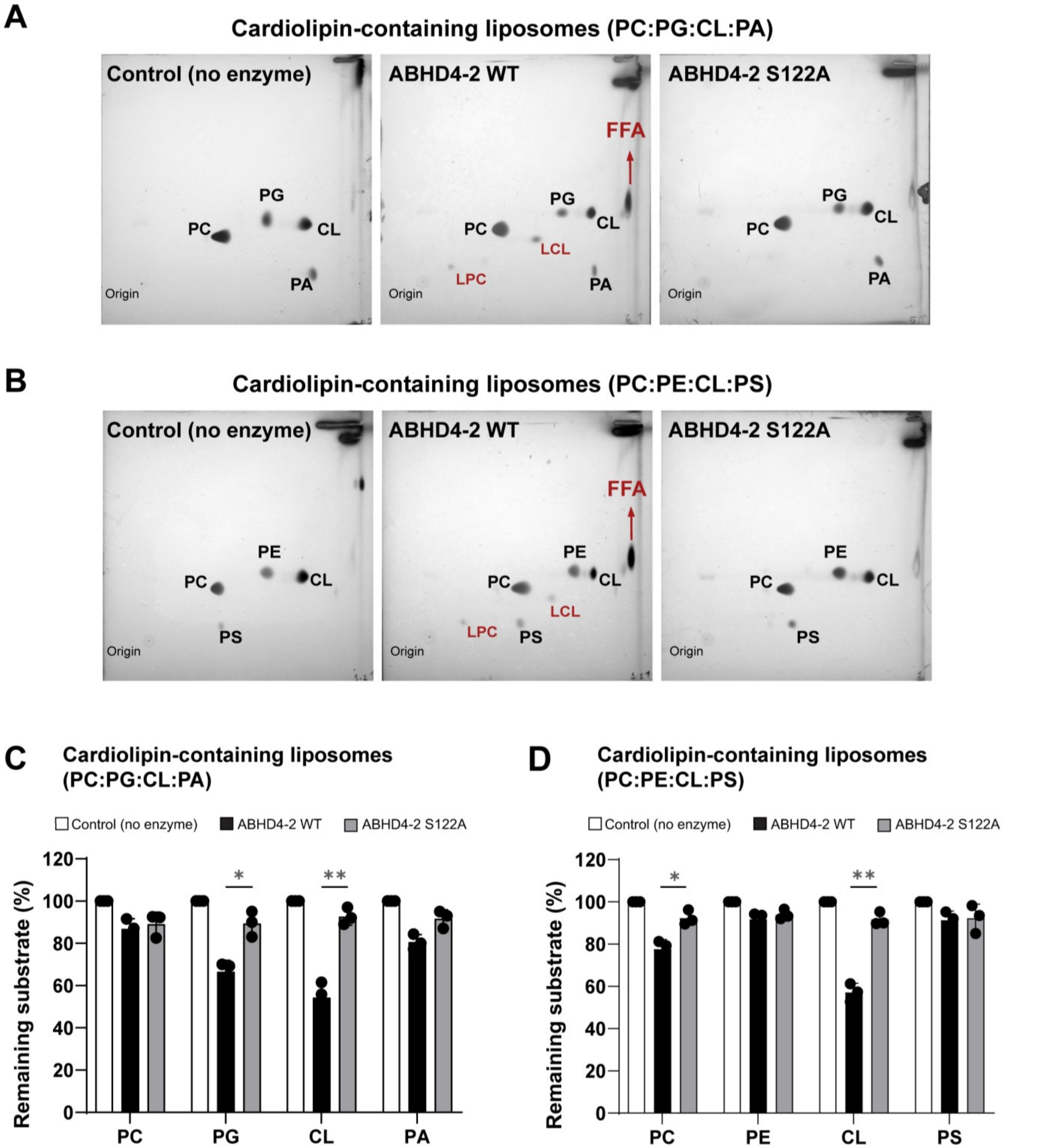

Supplemental Fig. S12. Liposome-based analysis of ABHD4-2 phospholipid hydrolysis in cardiolipin-containing membranes.

(A, B) Representative two-dimensional TLC of phospholipid vesicles containing PC, PG, CL, and PA (A) or PC, PE, CL, and PS (B), incubated with 2 µg recombinant ABHD4-2 WT or the catalytic mutant S122A for 2.5 h; no-enzyme controls were run in parallel. Lipids were resolved by two-dimensional TLC and visualized by copper sulfate charring. ABHD4-

2 WT generated LCL, LPC, and FFA in CL-containing vesicles; these products were absent in reactions with the S122A mutant.

(C, D) Densitometric quantification of the remaining phospholipid substrates, normalized to the corresponding no-enzyme control (100%). In panels C and D, white bars represent the no-enzyme control, black bars represent ABHD4-2 WT, and gray bars represent ABHD4-2 S122A. CL was hydrolyzed to the greatest extent, whereas PG and PC were hydrolyzed to a lesser extent, and PA and PE showed modest, non-significant decreases. Data represent mean ± SD from three independent experiments (n = 3). Statistical significance between ABHD4-2 WT and S122A was assessed by two-way ANOVA with Šídak's multiple comparisons test (**P* < 0.05, ***P* < 0.01).

PC, phosphatidylcholine; PE, phosphatidylethanolamine; PG, phosphatidylglycerol; PS, phosphatidylserine; PA, phosphatidic acid; CL, cardiolipin; LPC, lysophosphatidylcholine; LCL, lysocardiolipin; FFA, free fatty acid.

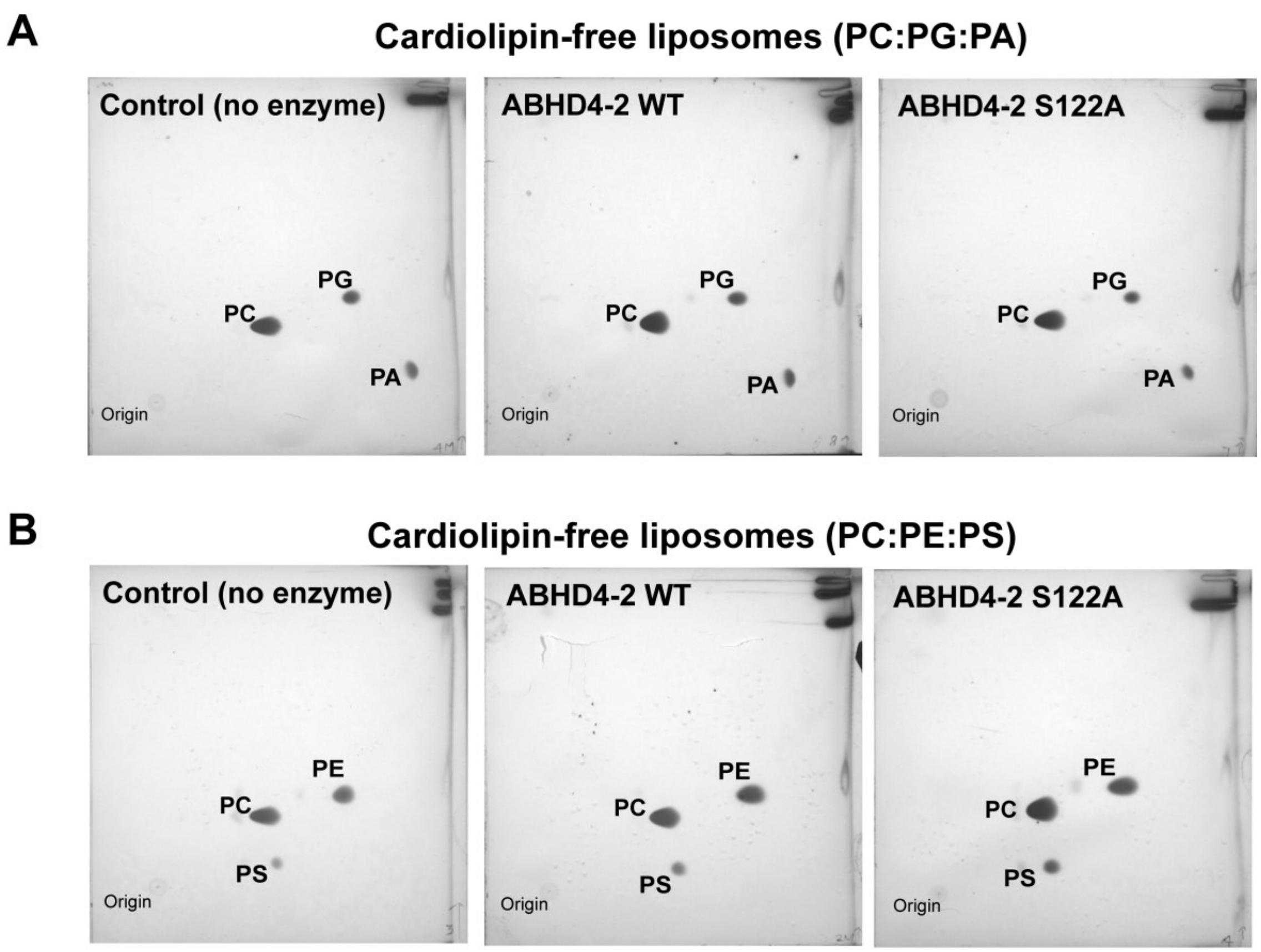

Supplemental Fig. S13. ABHD4-2 shows no detectable phospholipase activity in cardiolipin-free liposomes.

(A, B) Representative two-dimensional TLC of cardiolipin-free phospholipid vesicles containing PC, PG, and PA (A) or PC, PE, and PS (B), incubated with 2 µg recombinant ABHD4-2 WT or the catalytic mutant S122A for 2.5 h; no-enzyme controls were analyzed in parallel. Lipids were resolved by two-dimensional TLC and visualized by copper sulfate charring. No detectable FFA or lysophospholipid products were observed in reactions containing either ABHD4-2 WT or ABHD4-2 S122A. Representative images from three independent experiments are shown.

PC, phosphatidylcholine; PE, phosphatidylethanolamine; PG, phosphatidylglycerol; PS, phosphatidylserine; PA, phosphatidic acid.

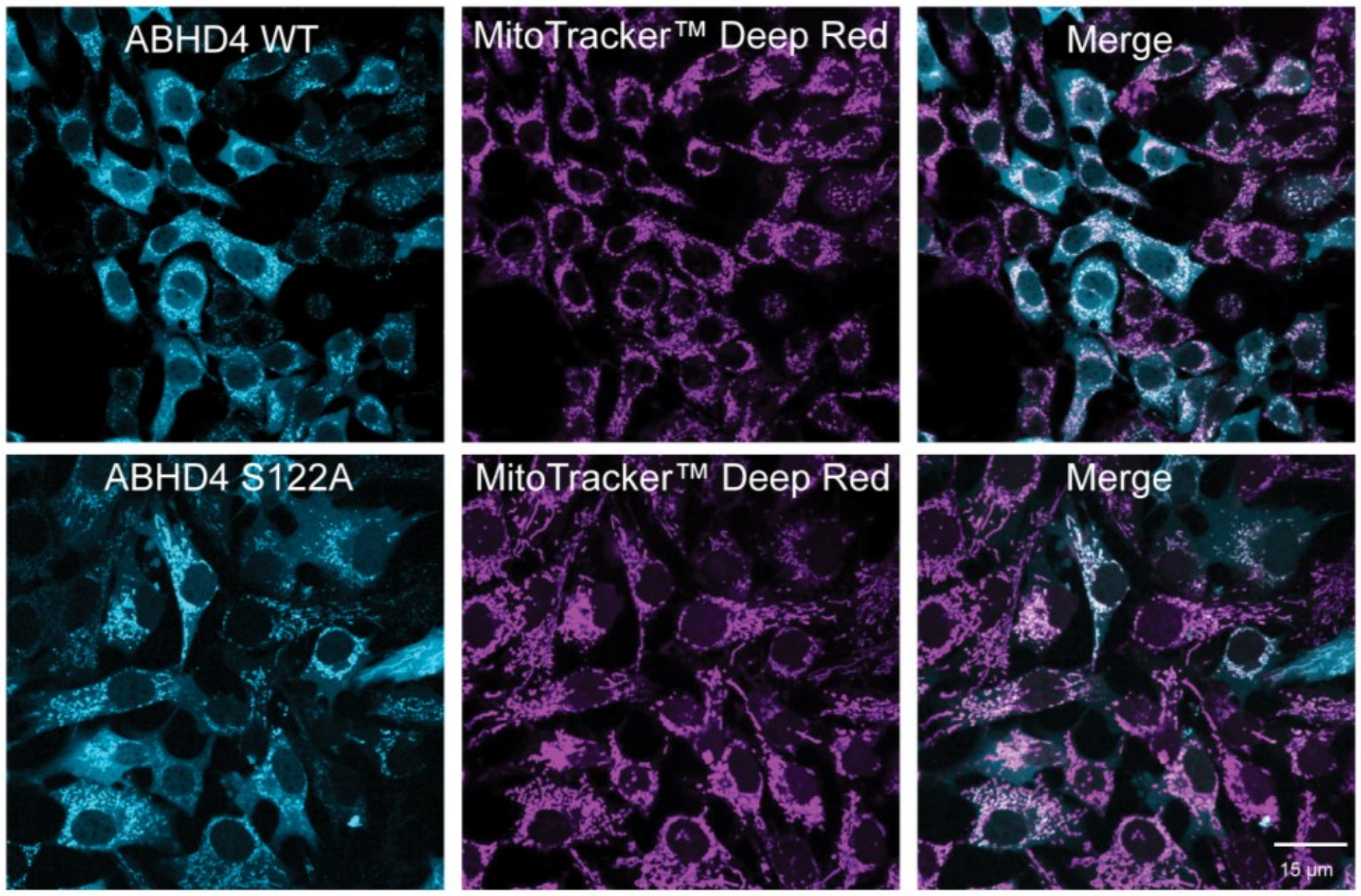

Supplemental Fig. S14: Mitochondrial localization of ABHD4-2 in brown adipocytes.

Confocal images of brown adipocyte stable cell lines expressing ECFP-tagged ABHD4-2 (WT) or the catalytic mutant S122A. Mitochondria were labeled with MitoTracker Deep Red. Both WT and S122A ABHD4-2 show colocalization with mitochondrial markers, indicating that mitochondrial targeting is independent of catalytic activity. Merged images show an overlap between ABHD4-2 and mitochondrial signals. Scale bar, 15 µm.
